# Towards Synoptic Water Monitoring Systems: A Review of AI Methods for Automating Water Body Detection and Water Quality Monitoring Using Remote Sensing

**DOI:** 10.3390/s22062416

**Published:** 2022-03-21

**Authors:** Liping Yang, Joshua Driscol, Sarigai Sarigai, Qiusheng Wu, Christopher D. Lippitt, Melinda Morgan

**Affiliations:** 1 Department of Geography and Environmental Studies, University of New Mexico, Albuquerque, NM 87131, USA; joshuadr@unm.edu (J.D.); rsa@unm.edu (S.S.); clippitt@unm.edu (C.D.L.); mhbenson@unm.edu (M.M.); 2 Center for the Advancement of Spatial Informatics Research and Education (ASPIRE), University of New Mexico, Albuquerque, NM 87131, USA; 3 Department of Computer Science, University of New Mexico, Albuquerque, NM 87106, USA; 4 Department of Geography, University of Tennessee, Knoxville, TN 37996, USA; qwu18@utk.edu

**Keywords:** surface water, water body detection, surface water extraction, water quality monitoring, remote sensing, artificial intelligence, computer vision, machine learning, deep learning, convolutional neural networks

## Abstract

Water features (e.g., water quantity and water quality) are one of the most important environmental factors essential to improving climate-change resilience. Remote sensing (RS) technologies empowered by artificial intelligence (AI) have become one of the most demanded strategies to automating water information extraction and thus intelligent monitoring. In this article, we provide a systematic review of the literature that incorporates artificial intelligence and computer vision methods in the water resources sector with a focus on intelligent water body extraction and water quality detection and monitoring through remote sensing. Based on this review, the main challenges of leveraging AI and RS for intelligent water information extraction are discussed, and research priorities are identified. An interactive web application designed to allow readers to intuitively and dynamically review the relevant literature was also developed.

## 1. Introduction and Motivation

Water is fundamentally necessary to all forms of life, and it is also the primary medium through which climate change impacts Earth’s ecosystem and thus the livelihood and wellbeing of societies [1]. While water covers about 71% of the Earth’s surface, only approximately 3% of the Earth’s water bodies are freshwater [2]. Climate change will bring unique challenges to these water bodies. Many rivers and streams are heavily dependent on winter snowpack, which is declining with rising temperatures and changing precipitation patterns [3]. Sea level rise is also impacting the continued quality and quantity of water supplies [4]. Both the quantity and the quality of freshwater systems are critical environmental features essential to increasing resilience in the face of climate change [5,6]. Resilience is defined here as the capacity of a system to absorb disturbance and still retain its basic function and structure [7]. Climate change will bring new disturbances in many forms, including increased pollution from wildfires, saltwater intrusion, and deteriorated water quantity resulting from prolonged drought [1,8]. It is critical that we gather, ideally automatically, as much information as possible about freshwater bodies and how they function in order to increase our capacity to respond to a changing climate. Rockström [5,6] and his colleagues conceptualize freshwater use and biogeochemical flows that threaten the integrity of freshwater (via pollution) as two of seven variables key to overall Earth system function. Each of these variables, they argue, can be thought of as having “planetary boundary”, a threshold that should not be crossed if we are to maintain the Earth in its current system state [5]. In this sense, the integrity and functioning of freshwater systems are essential not only in the local scale in which they provide critical ecosystem services; they also create a “safe operating space” for humanity as a whole, as we seek to achieve global solutions to the larger environmental challenges we face with climate change and associated stressors [6]. 

Responding to climate change challenge impacts on water resources requires adaptation strategies at the local, regional, national, and global scales. Countries are urged to improve their water resources management systems and to identify and implement “no regrets” strategies in order to be resilient to climate change [1]. The changing spatial and temporal patterns of surface water are important, in both practical and scientific terms, for water resources management, biodiversity, emergency response, and climate change [9]. More specifically, automated monitoring of water bodies is critical for adapting to climate change, water resources, ecosystem services, and the hydrological cycle, as well as for urban hydrology, which can facilitate timely flood protection planning and water quality control for public safety and health [10,11,12]. Accurate water quality monitoring is essential for developing sustainable water resource management strategies and ensuring the health of communities, ecosystems, and economies [13]. However, current knowledge of water quality is often disconnected in time and space across different measurement techniques and platforms that may fail to capture dynamic ecosystem changes. This disconnection indicates an inefficiency and redundancy in research and monitoring activities. A major challenge for water resource management is how to integrate multiple sources of water quality data and indices into usable and actionable information of environmental, social, economic, and infrastructural value [13,14]. 

Geospatial big data are leading to transformative changes in science (with the advent of data-driven and community science) and in society (with the potential to support the economy, public health, and other advances). Artificial intelligence (AI), especially its branches machine learning (ML), deep learning (DL), and computer vision (CV), are central to leveraging geospatial big data for applications in both domains. Remote sensing (RS) is the single largest source of geospatial big data and has increased dramatically in terms of both spatial and temporal resolution. This poses serious challenges for effective and efficient processing and analysis [15]. Meanwhile, recent advances in DL and CV have significantly improved research in RS and geosciences [16,17,18]. These advances, if integrated in creative and appropriate ways, host potential to enable the automated identification and monitoring of large-scale water bodies and water quality effectively and efficiently. 

In this article, we argue specifically that bridging research into extracting important water information (e.g., water body extent, water quality) from RS imagery will provide an important computational foundation for the development of smart, RS-enabled water resource management systems. We review a range of recent developments in the relevant fields that can be leveraged to support intelligent automation of water body extraction and water quality detection and monitoring through RS imagery. An accompanying interactive web application allows our readers to intuitively track scholars and publications covered in this review (the web app tool URL and its brief demo video link are provided in Appendix A).

### 1.1. Selection Criterion for Reviewed Papers and Brief Graphic Summary

**Figure 1:**
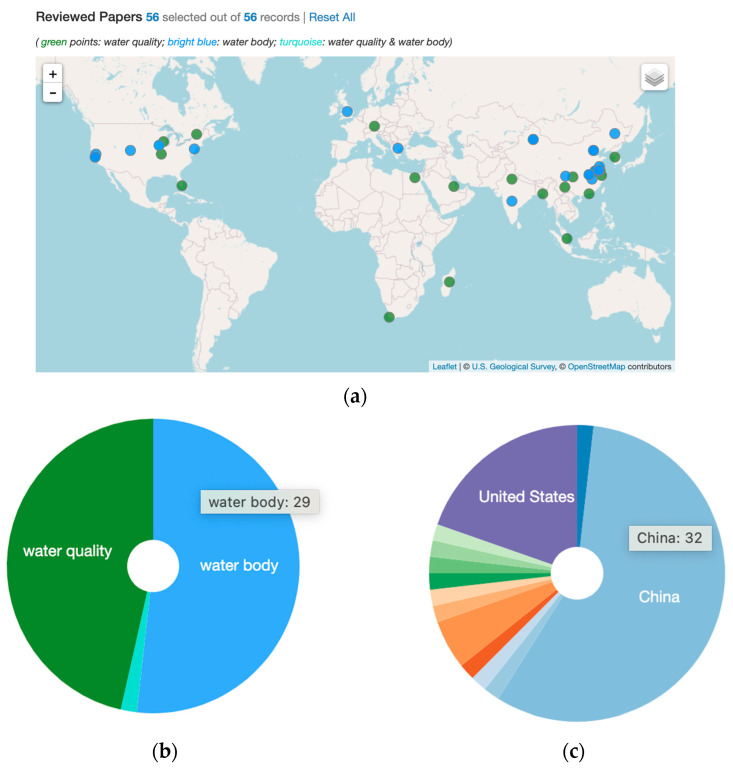

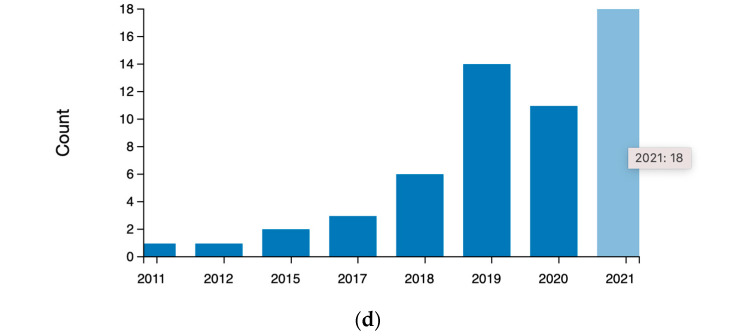
Geospatial distribution and simple statistics of the reviewed papers. Note that a freely accessible interactive version of the charts can be accessed via our web app tool (the web app tool URL and its brief demo video are provided in Appendix A). We can easily see that the major countries are China and the United States and that the number of published papers by year (2011 to 2021) has dramatically increased since 2018 and 2019. (**a**) Spatial distribution of reviewed papers based on the first author’s institution location. (**b**) Topic distribution (water body, water quality, both). (**c**) Country distribution. (**d**) Number of published papers by year from 2011 to 2021 on the relevant topics.

In the literature review process, we performed a systematic search on Google Scholar with the keywords and search strategy detailed in Table 1. In addition, our search was restricted to research articles published in English and in peer-reviewed journals or conference proceedings. For water body detection, we combined the water body keywords with some combination of the general keywords. The process for finding publications related to water quality was the same, only with the water quality keywords list. Beyond the keywords listed in this table, references (those cited in the papers we reviewed) cited by the keyword-identified papers were also retained. A total of 90 papers relevant to the topic of water body and/or water quality from RS imagery using AI/ML/DL/CV algorithms were identified. A total number of 56 highly relevant articles were identified by applying the following exclusion criteria: (1) papers related to plastic pollution and sewage/water treatment plants, (2) precipitation forecasting or groundwater detection (as it is not intuitive to detect groundwater from RS imagery), and (3) general land use classification. Figure 1 shows the spatial distribution and a simple statistics summary of the papers covered in this review, where (d) shows the number of published papers by year in the reviewed topics from 2011 to early 2022. 

### 1.2. Roadmap

Here, we provide a roadmap for the rest of the paper. Section 2 outlines the scope of this review and our intended audience. Section 3 is the core of the paper, focused on identifying important and recent developments and their implications to water body detection and water quality monitoring from RS imagery through the leverage of AI/ML/DL/CV. Here, we highlight recent advances in several subfields of AI that water domains and RS can leverage. Specifically, we provide general characteristics of the reviewed studies using word clouds (Section 3.1). We then examine and appraise key components of influential work in water body detection (Section 3.2) and water quality monitoring (Section 3.3). Section 4 starts with a brief summary (Section 4.1), followed with a discussion of key technical challenges (Section 4.2) and opportunities (Section 4.3). The paper concludes in Section 5.

To allow our readers to intuitively and dynamically review the relevant literature, we have developed a free-of-charge interactive web app tool (the web app URL and its brief demo video are provided in Appendix A). To provide background for readers (particularly those from water resources and RS) who are new to AI/ML/DL/CV, we introduce essential ML terms in Appendix B. As evaluation metrics are essential for measuring the performance of AI/ML/DL/CV models, we also provide an introduction to a set of commonly used evaluation metrics in Appendix C. In addition, as there are plenty of acronyms in this paper, we provide a full list of abbreviations right before the appendices.

## 2. Audience and Scope

It is important to know where water is and how its extent and quality are changing over time in a quick and accurate manner. Water quality is a key issue in water supply, agriculture, human and animal health, and many other areas [19]. Impaired water quality can be caused by natural disasters, but the most common cause is anthropogenic pollution. Pollutants, excessive nutrients from fertilizers, and sediment (e.g., from soil erosion) are carried into local lakes and rivers via runoff from urban or agricultural areas [19,20]. The quality of water varies from places and from time to time [19]. Affected surface waters are present in RS imagery and can be identified with the help of computational techniques such as ML. *To make near real-time intelligent water body detection and water quality monitoring possible, we need to first detect extent of water bodies from RS imagery, from which volume can be computed, and then recognize their corresponding water quality, eventually linking the two to allow water quality monitoring*.

Environmental nonprofits, government agencies, and water managers need access to this type of integrated spatial–time series of water body and water quality information to see how local water resources are changing and plan for future drought conditions. Collective detection and monitoring of water bodies and their associated water quality has applications for human health, as well as to private-sector industries including timber, agriculture, recreation, and tourism. Public policy planners need to be better informed as they make environmental preservation and restoration decisions based on changing water availability, and with this data we can be better equipped to monitor water quality that can quickly change due to floods, hurricanes, or human-caused pollution, *and yet, to date, water body detection and water quality monitoring research has been historically separate and does not focus enough on producing intuitive, operational products*.

Building on the long-term interest in ML and CV within the RS community, the main goals of this review paper are to (1) survey recent advances in water body detection and water quality monitoring from RS data using AI to identify commonly cited challenges in order to provide suggestions for new research directions, and (2) move towards automated, synoptic water quantity and quality monitoring to inform more robust water resource management. 

This systematic review is relevant to multiple research domains, including, but not limited to RS, geographic information science, computer science, data science, information science, geoscience, hydrology, and water resource management. This paper does not attempt to review the application of RS to water resources and hydrology more generally; for recent reviews of these topics, see [13,21,22,23,24]. A survey of DL applications in hydrology and water resources can be found in [25]; a survey of AI in the water domain can be found in [26]; and a survey of water quality applications using satellite data solely focused on ML can be found in [27]. This review focuses on investigating recent AI methods, including its branches ML, DL, and CV, for water information extraction (specifically water body detection and/or water quality monitoring) from RS imagery. Our review has a narrowed scope in water resources and hydrological research, but a wider and deeper scope in terms of AI methods and metrics used to assess models in both water body detection and water quality research. *By integrating both domains, we hope to develop a basis for effective computational frameworks for intelligent water monitoring systems using RS and AI.*

## 3. The State of the Art: Advances in Intelligent Waterbody Information Extraction

### 3.1. General Characteristics of the Reviewed Studies

**Figure 2:**
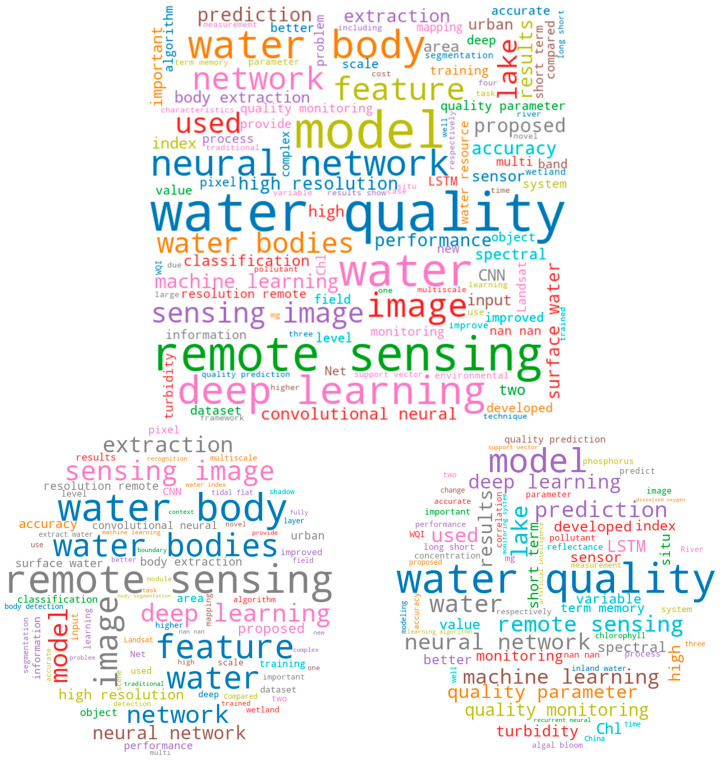
Word cloud visualization of all the reviewed papers (**top**), water body papers (**bottom left**), and water quality papers (**bottom right**). Note that the word clouds are generated from paper titles, abstracts, and keywords. The word clouds provide an informative (general and specific) focus of each set of the papers. For example, both water body and water quality papers share the focus on RS, DL, and neural networks (NN). We can also see that water body extraction tasks tend to focus on the use of convolutional neural networks (CNN), whereas for water quality modeling the use of long short-term memory (LSTM) networks is more prevalent. We can also see that there are specific, unique keywords for water quality, such as “turbidity”, “chl”, and “algal bloom”.

Note that we only included and reviewed the papers that use both RS and AI/ML/DL/CV for water body and/or water quality detection (that is, the number of papers cited in our reference section is much larger than the number of papers we review in this Section 3). A word cloud visualization of the titles, abstracts, and keywords of the reviewed 56 papers are provided in Figure 2, where the top figure indicates the word cloud for all reviewed papers. The bottom left word cloud is for reviewed water body papers, and the bottom right for reviewed water quality papers.

As we can see from the word cloud for both water body extraction and water quality (see the top word cloud in Figure 2), “remote sensing”, “deep learning”, “prediction”, “classification”, “extraction”, “machine learning”, “water body”, “water quality”, and “convolutional neural network” are prominent concepts and words captured by the word cloud. Our focus is on studies that use RS for water body extraction and water quality monitoring, so many of the keywords are to be expected. However, it is perhaps surprising to see DL featured so prominently given that the shift from ML to DL models is a relatively recent phenomenon.

When we separate the keyword word clouds (see the bottom two word clouds in Figure 2), this trend becomes clearer. Deep learning is much more common in water body extraction, whereas in the word cloud for water quality monitoring, “neural network” and “machine learning” are about the same size. Additionally, in the water body extraction word cloud, “remote sensing” is featured much more heavily than it is in the water quality extraction literature. In our review, the water quality papers often involved other types of data, including in situ sensors or smaller RS devices (not satellites), whereas the water body extraction literature is dominated by RS imagery. This is related to the scale of projects in the two domains: water body extraction is usually undertaken across large spatial scales, whereas the water quality monitoring literature is still focused on smaller, often individual, bodies of water. This points to a future research direction in the water quality literature that we touch on in our review paper: we need to scale up water quality estimation using RS imagery by matching it with ground-truth water quality measurements.

Table 2 and Table 3 provide a brief summary of the methods used for water body detection and water quality monitoring, elaborated in Section 3.2 and Section 3.3, respectively. The general characteristics summarized by machines (i.e., the word clouds in Figure 2) align with the literature; convolutional neural network (CNN) models are indeed applied much more frequently for water body detection, and long short-term memory (LSTM) models are often used for water quality monitoring. The evaluation metrics used in the reviewed articles were also summarized and are provided in Table 2 and Table 3 (a brief explanation of each metric is in Appendix C).

### 3.2. Recent Advances in Water Body Detection Using AI

From our systematic review (including Table 2), we provide a brief summary here about the recent advances in water body detection based on AI. (1) The most common satellite platforms were Landsat, GaoFen, Zi Yuan, WorldView, and Sentinel, although there were some manually annotated datasets. The use of UAVs and DEMs were noted but were not as common. (2) Precision, recall, overall accuracy (OA), F1-score, kappa, and intersection over union (IoU) are the most popular evaluation metrics for water body detection since it is mainly a classification task. (3) Convolutional neural networks (CNNs) are normally compared to normalized difference water index (NDWI) or another index-based method, some form of “shallow” ML model (e.g., random forest (RF), support vector machine (SVM)), or other CNN architectures). Below, we provide a more detailed review of the methods used for water body detection. As Table 2 and word clouds (see Figure 2) indicate, the dominant methods used in water body detection with AI are CNNs (Section 3.2.1). Beyond CNN-based methods, there are other methods including CNN hybrids (Section 3.2.2), artificial neural networks (ANN), multilayer perceptrons (MLP), dense neural networks (DNN), other DL methods (Section 3.2.3), and “shallow” ML based methods (Section 3.2.4).

#### 3.2.1. CNN-Based Water Body Detection

CNN-based models are the dominant methods for water body detection, but each of them have addressed different challenges posed in water body detection from RS imagery. Based on our review, we identify the following five groups of use cases: (1) Addressing limitations of index-based methods; (2) sharpening blurred boundaries caused by CNNs; (3) Addressing spatial and spectral resolution challenges, which covers those methods that are able to recognize water body across scales, at multiple resolutions, from very high-resolution imagery, and/or integrating bands beyond RGB channels to use for CNN model training; (4) Robust detection of small/slender/irregular-shaped water bodies; (5) Others.

1. Addressing limitations of index-based methods:

Index methods (e.g., NDWI) are rule-based and fail to take advantage of context information. CNNs can overcome this, although they often blur boundaries in segmentation tasks because of the convolution operation [34]. A DenseNet was used in [43] for water feature extraction and the authors compared its performance with NDWI and several popular CNN architectures. While NDWI methods are quick, they are not as accurate as CNNs. The authors showed that DenseNet performed the best at distinguishing water from shadows and clouds. However, the authors argue that clouds often occlude optical imagery, so one way to improve their method is to combine it with microwave RS imagery.

The authors in [31] pointed out that index methods require careful calibration and that indices differ from place to place. They also suffer from false positives (from snow, ice, rock, shadows, etc.) and vary in different weather conditions (e.g., clouds). To overcome those limitations of index-based methods, the authors of [31] developed DeepWaterMap, which can classify water with high accuracy, even distinguishing it from snow, ice, shadow, and clouds. DeepWaterMap is able to classify land classes that are often misclassified as water (or vice versa); thus, it minimizes false positives during the classification process. Most importantly, the DeepWaterMap model also works across different terrains and in different weather conditions, although it is still affected by clouds. The same authors released a second version of the model, DeepWaterMap v2, in [40]. The major improvement from v1 is that the new version allows users to input large RS scenes without the need for tiling, and the authors made their network run efficiently with constant memory at inference time. This model should theoretically work across different sensor platforms as long as they have the visible, near-infrared, and shortwave infrared 1 and 2 bands, but will still sometimes classify clouds as water.

2. Sharpening blurred boundaries caused by CNNs:

CNN-based methods can overcome the limitations of index-based methods, as reported above in group (1) [34], but they often blur boundaries in segmentation tasks because of the convolution operation. To sharpen water body detection boundaries, in [34], a restricted receptive field deconvolution network (RRF DeconvNet) and a new loss function called edges weighting loss were proposed. However, the authors needed to retrain the entire network (which is very computationally expensive) instead of using transfer learning (TL). 

Apart from blurring pixel boundaries, CNNs generally require many training parameters and very large training datasets to be successful. A novel convolution–inception block in a network, called W-Net, was proposed in [48], to extract water bodies from RS imagery. W-Net is able to train on fewer images compared with other CNN models and still extract water bodies accurately, and the authors pointed out that less computations are necessary due to use of inception layers. W-Net outperformed other CNN architectures, although the authors still needed to go through the time- and labor-intensive process of creating a dataset of manually annotating images.

3. Addressing resolution and band related challenges

High-resolution optical RS imagery allows for much finer detail in surface water body extraction. However, clouds and their shadows are often present in optical RS images [78]. The shadows (e.g., cloud shadows and building shadows) and water bodies share a very similar appearance in optical RS images. Therefore, water body extraction is not an easy task in the optical high-resolution RS images due to the limited spectral ranges (including blue, green, red, and near-infrared bands) and the complexity of low-albedo objects (cloud shadows, vegetation, and building shadows). Higher spatial resolution imagery often comes at the cost of less spectral channels and thus makes it difficult to extract features from complex scenes. To address this problem, a dense local feature compression (DLFC) was proposed [52] to extract bodies of water from RS imagery, and their DLFC outperformed other state-of-the-art (SOTA) CNNs, as well as an SVM and NDWI thresholding. Their results demonstrated that the DLFC is good at extracting slender water bodies and distinguishing water bodies from building shadows using multisensor data from multiple RS platforms.

TL and data augmentation (see Appendix B) are used in [37] to extract water bodies from satellite imagery. The authors showed that a CNN can outperform NDWI and an SVM in water body detection when the input data is very high resolution. There are tradeoffs, however, and the authors reported that the difficulty of hyperparameter tuning is one downside to using a CNN. A water body extraction NN, named WBE-NN, was proposed in [45] to extract water bodies from multispectral imagery at multiple resolutions while distinguishing water from shadows, and performed much better than NDWI, an SVM, and several CNN architectures. A self-attention capsule feature pyramid network (SA-CapsFPN) was proposed in [49] to extract water bodies from satellite imagery of different resolutions. SA-CapsFPN is able to recognize bodies of water across scales and different shapes and colors, as well as in varying surface and environmental conditions, although it is still entirely dependent on optical imagery as input to the CNN. 

The novel MSResNet proposed in [46] learned from a large dataset of unlabeled RS imagery. MSResNet, in addition to being able to extract water bodies in an unsupervised manner, is able to recognize water bodies at multiple resolutions and of varying shapes. However, their network cannot distinguish water bodies from farms and barren areas. In addition, the CNN-based model name FYOLOv3, proposed in [51], is able to detect tidal flats at different resolutions. However, it does depend on a manually selected similarity threshold that introduces some subjectivity.

RGB band imagery is the primary focus in substantial research for water body extraction, but many more bands are available in RS imagery. A multichannel water body detection network (MC-WBDN) was created in [47], which fused the infrared and RGB channels and used them as input data for their CNN architecture. They demonstrated that when multispectral data is used, model performance for water body detection is increased and the model is more robust to lighting conditions. The proposed model MC-WBDN is much more accurate than index-based methods such as NDWI, modified NDWI (MNDWI), and normalized difference moisture index (NDMI). MC-WBDN also outperforms other SOTA architectures such as U-Net and DeepLabV3+ for water body detection tasks. However, this method still relies on preprocessing data to make sure each input image is the same shape and free of clouds.

4. Robust detection of small/slender/irregular-shaped water bodies

Small water bodies are hard to extract from RS imagery. In [33], the authors designed a CNN (named SAPCNN), which is able to extract high-level features of water bodies from input data in a complex urban background. NDWI and SVMs cannot distinguish between water and shadows and their architecture’s performance partly relies on visual inspection. Ref. [53] utilized a modified DeepLabv3+ architecture to extract bodies of water at different scales. Their focus is on extracting water bodies in urban RS images. Their network performed well on small bodies of water, but the model has problems identifying many of them because they were not properly annotated. 

Mask-region-based CNNs (R-CNNs) have demonstrated success in detecting small and irregular shape water bodies. Song et al. (2020) [41] employed an R-CNN for water body detection from RS imagery, and their model outperforms many traditional ML models in identifying small water bodies and bodies of water with differing shapes. However, it is still difficult to deploy a trained NN model into a usable, production-ready form for water mapping applications. The authors reported that using NN output to create and update a vector map of water resources for stakeholders is challenging.

Yang et al. (2020) [42] also used a mask R-CNN to automate water body extraction. The authors argued that this allows them to avoid manual feature extraction in complex RS imagery. They segmented small water bodies and bodies of water with irregular shapes, although their methods suffer from poor IoU accuracy. This is primarily due to a small training set, for which DL models are ill-suited, and resulted in their models having problems identifying multiple bodies of water in RS images.

A self-attention capsule feature pyramid network (SA-CapsFPN) was proposed in [49] to extract water bodies from satellite imagery. SA-CapsFPN is able to recognize bodies of water across scales and different shapes and colors, as well as utilizing different information channels. The novel MSResNet proposed in [46], learnt from unlabeled large RS imagery, is also able to recognize water bodies at multiple resolutions and of varying shapes; however, their network cannot distinguish water bodies from farms and barren areas.

A dense local feature compression (DLFC) was proposed in [52] to extract bodies of water from RS imagery, and their DLFC outperformed other SOTA CNNs, as well as an SVM and an NDWI. Their results demonstrated that the DLFC is good at extracting slender water bodies and distinguishing water bodies from building shadows using multisensor data from multiple RS platforms.

5. Others

Extracting water bodies from RS imagery quickly and reliably is still a difficult task. Based on U-Net, [50] developed a new model called SU-Net to distinguish between water bodies, shadows, and mixed scenes. However, the authors only focused on water body extraction in urban areas and only used RGB information during the extraction process. While SU-Net performed better than an SVM and classic U-Net, it suffered when extracting water bodies from RS imagery with high reflectivity or that contained aquatic plants.

Wetlands are important ecosystems because they can keep flooding at bay and store carbon; however, they are threatened by development, climate change, and pollution. For the task of identifying wetlands, [44] combined RS imagery with hydrological properties derived from digital elevation models (DEMs) to identify wetlands. They showed that an RF performs as well as a CNN, although both models had issues distinguishing roads and trees from wetlands. This is perhaps due to their small training set. To improve performance, the authors argued that larger datasets with finer labels should be created for wetland detection.

Substantial water body detection work has focused on water bodies in urban and inland settings. Very few focus on tidal flat extraction, where sediment levels are high and the boundary of the water body itself is blurry. A CNN model called FYOLOv3 was proposed in [51], where the authors compared their model to NDWI, an SVM, a maximum likelihood classifier, U-Net, and YOLOv3. FYOLOv3 performed the best and is able to detect tidal flats at different resolutions; however, it depends on a manually-selected similarity threshold during the training process, which is a source of subjectivity.

Large sets of unlabeled water body data are available and easy to acquire, and semantic segmentation networks cannot recognize different water body shapes. A recent, very novel encoder–decoder CNN architecture named MSResNet, proposed in [46], is able to overcome those limitations. MSResNet is able to learn from unlabeled data and can also recognize water bodies of varying shapes and at multiple resolutions. However, even though their network outperforms other SOTA architectures without supervised training, their network has some issues categorizing water bodies, farms, and barren areas.

#### 3.2.2. CNN Hybrid-Based Water Body Detection

CNNs are the SOTA models in water body extraction tasks (detailed in Section 3.2.1 above); however, their output and decisions for why they make the predictions that they do are largely a black box. Recent studies have integrated CNNs with some ML models. Interpretability was improved by using a CNN and SVM in parallel to classify wetland water bodies [39]. Wetlands are difficult/complex to identify in high-resolution satellite imagery with any single ML model. Hybrid models have shown promise in a process called decision fusion. Here, the authors pick a decision fusion threshold value by performing cross-validation on the CNN to see when it is sure or not. They then use this threshold value for the decision fusion predictions (e.g., when the CNN is not that sure, they defer to the SVM). However, the authors did not explain why they used an SVM and not some other ML model. The classifier used in [32] combines a CNN with a logistic regression (LR) model to extract water bodies. The authors emphasized that traditional ML methods for water body extraction need multispectral data and rely on lots of prior knowledge. Thus, those ML-based methods would not generalize well to different tasks. The authors also argue that single-band threshold methods are subjective. Their results demonstrated that the hybrid CNN-LR model works better than an SVM, an ANN, and other CNNs. However, their method requires segmented RS images as input.

How to accurately extract water bodies from RS images, while continuously updating the surface water maps, is an active research question. Index methods and active contour models are popular methods for water body detection tasks but are sensitive to subjective threshold values and starting conditions. Deep U-Net model was proposed to be used with a conditional random field (CRF) and regional restriction to categorize water versus non-water in satellite images [36], while reducing the blurring of edges that often occurs from CNNs for image segmentation. Although this network is highly accurate, it takes a lot of data and computation power to train. Training ML models at a single scale in single channels can cause errors when generalizing to other scales or types of RS data. Multiscale RS imagery was used with DeepLabV3+ and a CRF for water body segmentation [38]. This approach works well for training models on data from different scales, and they concluded that CNNs and CRFs together extract more accurate water boundaries at both large and small scales than CNNs alone.

#### 3.2.3. ANN, MLP, DNN, and Other DL-Based Methods for Water Body Detection

An NN architecture called a local excitatory globally inhibitory oscillator network (LEGION) is used in [28], where the authors compared the results of LEGION trained on NDWI and spectral information, respectively. In addition, they employed object-wise classification, instead of pixel-based classification used in most other work. The authors reported that the network is very computationally expensive.

Different methods of water body extraction work (or do not work) in different areas/terrain types. Each needs subjective thresholds and/or hand-crafted features. In addition, generating large sets of labeled data is difficult and expensive, as high-dimension RS data is difficult to analyze. Objects such as shadows, clouds, and buildings are hard to distinguish from water bodies. In [29], the authors used an autoencoder for unsupervised training and concluded that their results are more accurate than for an SVM and traditional NN.

Huang et al., 2015 [30] pointed out that not many people have focused on water body detection in urban settings. This is a problem because water bodies often look similar to shadows due to buildings at certain times of the day in optical imagery. The authors employed an extreme learning machine (ELM), an SVM, a tree bagger (TB), and an RF to detect water bodies. The authors reported that the RF and TB performed much better than the SVM and ELM. However, their method depends on optical imagery with subjective thresholds set through trial and error. Specifically, their method depends on subjective threshold values in NDWI, normalized difference vegetation index (NDVI), and morphological shadow index (MSI). 

Ref. [10] compared MLP, NDWI, and a maximum likelihood model for water body classification and showed that MLP performed the best. However, the maximum likelihood model could not recognize small bodies of water and thin rivers, whereas NDWI was not able to distinguish seawater from land. The MLP could identify small bodies of water better, but the analysis depended on visual assessment.

#### 3.2.4. “Shallow” ML-Based Water Body Detection

Although most of the recent methods for water body detection used DL and/or deeper neural networks (Section 3.2.1, Section 3.2.2 and Section 3.2.3), a few studies used only “shallow” ML methods (e.g., RF and SVM). In [35], the authors used band methods (where slope, NDVI, and NDWI were added as three secondary bands to integrate extra information into ML training), and then applied an SVM, a decision tree (DT), and an RF to analyze multiband RS data for water body extraction in the Himalayas. However, while their models worked well for flat and hilly terrain, they had to parse out high elevations and snow in this method (which involves extra preprocessing and limits when/where their method can work with optical data). The authors ran different experiments to analyze which input bands (NDWI vs. individual input bands from Landsat data) worked the best but could only compare results visually. The authors concluded that adding single secondary bands is better than adding multiple in most ML algorithms except for NNs.

Sentinel-1 data and four different ML models (K-nearest neighbors classifier (KNN), fuzzy-rules classification, Haralick’s textural features of dissimilarity, Otsu valley-emphasis) were employed to classify water bodies in [54]. It involved many different ML methods in tandem (i.e., the output of one ML model was fed into other processing steps), which complicates interpretability. This method did not have very high accuracy and did not work well in flooded regions, near buildings, and in the presence of aquatic vegetation. However, it was an important attempt to use synthetic aperture radar (SAR) data, which is rare in water body detection literature.

### 3.3. Recent Advances in Water Quality Monitoring Using AI

From Table 3, we identify the following trends in the use of AI for water quality monitoring research: (1) Water quality monitoring differs from water body detection in that it is formulated as both a classification and a regression task. Because of this, recurrent neural networks (RNNs), long short-term memory (LSTMs), and gated recurrent units (GRUs) are much more prevalent in the water quality literature. (2) Accuracy, precision, and recall are common metrics, as are some variations of mean squared error (MSE) and R^2^. (3) It is important to note that while water body detection papers describe integrating multiple data sources into one analysis, this practice is much more common in water quality monitoring research. This primarily takes the form of trying to match up water quality parameters from time series data or water samples to optical satellite RS imagery. In water quality monitoring, it is much more common to utilize Internet of Things (IoT) sensors, smaller probes such as unmanned aerial vehicle (UAVs) and stationary hyperspectral imagers, as well as government and private water quality time series data. (4) Some studies do not compare their model to any other models (detailed in Table 3), making it difficult to fully assess their methodologies. 

Below, we provide a more detailed review of the methods used for water quality detection and monitoring. As our manual investigation (see Table 3) and machine summary (word cloud, see Figure 1) indicate, the dominant methods used in water quality detection with AI are LSTMs (Section 3.3.1) and ANNs, MLPs, DNNs, and other DL methods (Section 3.3.5). Beyond LSTM and ANN-based methods, there are other methods including LSTM hybrids (Section 3.3.2), CNN-based methods (Section 3.3.3), and “shallow” ML-based methods (Section 3.3.4).

#### 3.3.1. LSTM-Based Water Quality Detection and Monitoring

Algal blooms cause serious harm to human and animal health and can damage both environments and economies. Various factors lead to algal blooms and gathering the data necessary to predict them is time- and cost-intensive. ML models can provide advanced warning for these events by taking into account time series data of basic water quality parameters. A linear regression model was compared with an MLP, an RNN, and an LSTM to predict harmful algae blooms in dammed pools from several rivers [57]. While the LSTM model was the most accurate overall, for several of the dammed pools that the authors tested, a least-squares regression model outperformed the LSTM. This casts doubt as to how the LSTM model generalizes and if it is worth the added complexity.

Water pollution is becoming an increasing problem because of rapid rates of development and urbanization. Large amounts of water quality parameters can be taken via IoT sensors, and DL techniques are well suited to finding patterns in the large quantity of data. An LSTM was used to predict future values of different water quality parameters [60]. Most importantly, the authors only used single-dimensional inputs and outputs (i.e., a 1D time series of dissolved oxygen as an input to predict dissolved oxygen at some time in the future). While the results were good, the authors noted that the architecture would benefit from training on multiple time series at the same time. The authors reported that long-term predictions on the order of 6 months into the future did not work well. Beyond monitoring water for different levels of pollutants, it is also important to find the sources of pollutants when they are identified. Cross-correlation was used to map pollutants to different water quality parameters [58]. They then used an LSTM to match pollutants to nearby polluting industries using the highly correlated water quality parameters.

Similar to LSTMs, RNNs have been demonstrated to be accurate for times series prediction but are also often criticized for being difficult to interpret. Meanwhile, process-based ecological models, although deterministic, fail to capture patterns at longer time scales. A process-based model was integrated with an RNN to better align predictions of phosphorus levels in lakes to eliminate outlier predictions. Constraining NN output with physics-based models better aligns their predictions with ecological principles [68].

Rapid development has led to decreased water quality. In [70], water quality parameters can be used to both classify the current water quality index and predict future water quality index states. However, the authors separately compared DL models for water quality prediction and ML models for water quality classification, making the methods not directly comparable. A nonlinear autoregressive neural network (NARNET), a type of ANN, performed better than an LSTM at predicting the water quality index, while an SVM performed better than other traditional ML models for classification.

#### 3.3.2. LSTM Hybrids Water Quality Detection and Monitoring

To further improve model performance, a few recent studies have integrated other models with LSTMs. Water scarcity and drought are increasingly significant environmental challenges. Increased development is leading to worsening water pollution. Predicting the water quality from time series data is essential, but traditional ML models fail to capture long-term temporal patterns. This causes them to make false predictions in water quality monitoring applications. An RNN–Dempster–Shafer (RNN–DS) evidence theory hybrid model was used to make sense of multiple input time series of different time scales [63]. While evidence theory did make the predictions more stable, longer-term predictions did not work very well, even with the improvements to the model. The authors pointed out one possible reason might have been not taking spatial correlations between water quality parameters into account.

Economic development and urban growth have posed water quality issues. Wavelet domain threshold denoising (WDTD) and wavelet mean fusion (WMF) were used to analyze the output of LSTM predictions for multiple water quality parameters [65]. While multiple wavelet basis functions were used to analyze predictions, the LSTM was not compared to any other models in this analysis. The authors noted that not having enough observations was a limitation while training their LSTM model.

Mangrove wetlands provide habitats for many different types of animal species in addition to preventing coastal erosion. More recent research has focused on monitoring the water quality in these environments to assess the health of coastal ecosystems. Using water quality and meteorological time series data, three different submodels were used for each water quality parameter at different time intervals and fused their output predictions [66]. The authors tested this setup with a DNN, a gated recurrent unit (GRU), and an LSTM model. While the LSTM performed the best, the authors reported that the model is not very reusable or user-friendly.

Collecting and analyzing water samples is expensive, time-consuming, and labor-intensive. Thus, many researchers choose to use sensors to remotely monitor water quality parameters, but the number of parameters they can record are often limited. Ref. [69] used a submerged multiprobe sensor to monitor several important water quality parameters over the course of 1 year. They found that a CNN–LSTM model performs better than standalone DL models and traditional ML methods for predicting water quality parameter values; however, the authors did not use a validation set during NN training and the hybrid model was able to quickly learn the training and testing set data distributions.

#### 3.3.3. CNN-Based Water Quality Detection and Monitoring

CNNs are the dominant architecture for water body detection (Section 3.3.1 and Section 3.3.2) but are not used as widely for water quality. Here, we review two very interesting but effective CNN-based methods. In situ water quality measurements work really well but are very expensive. In addition, things such as total nitrogen and phosphorus, biological oxygen demand, and dissolved oxygen are hard to measure from satellites because they have weak optical properties. A CNN was used in [59] and showed that TL beats out traditional ML models when classifying water quality from RS imagery. However, their dataset was very small, and their focus was narrow (specifically, only two lakes in China, no rivers or coastal waters covered). Water bodies are often polluted, or their quality is affected from far away and thus it is difficult to identify and report on water quality. Methods for estimating water quality at scale are essential. Turbidity can be a proxy for total suspended solids (TSS) and suspended sediment concentration (SSC), so [72] used image detection and then applied edge detectors to UAV images of water. They employed CNNs to detect changes in water color and utilized this to approximate quality. They showed that image-based turbidity detection is as accurate as actual turbidity meters, but more importantly represents a very promising method for monitoring water quality at greater spatial scales.

#### 3.3.4. “Shallow” ML-Based Water Quality Detection and Monitoring

Remote water bodies are hard to monitor for water quality. A simple NN architecture was designed to estimate several water quality parameters (i.e., chlorophyll-a, turbidity, phosphorus) both before and after an ecosystem restoration project during both the dry and wet seasons [55]. Importantly, their predictions, using seven different input bands for training the NN, were very close to the actual values.

Finding what data to input into an ML model for water quality monitoring is neither easy nor straightforward. Different indices are sensitive to different areas and varying weather and lighting conditions. To address this problem, [71] first correlated water quality parameters to different RS bands. These correlations were then used to test four ML models and their ability to predict a water quality index. Their R^2^ statistics were not high, though.

#### 3.3.5. ANN, MLP, DNN, and Other DL-Based Methods for Water Quality Detection and Monitoring

Climate change is making droughts and water shortages increasingly worse in arid regions. It is thus important to develop methods and systems for intelligent and efficient monitoring of the water resources in those regions. A water quality index for arid regions was proposed in [56] and attempted to find which bands and spectral indices are related to that water quality index. In situ water quality sampling is labor- and cost-intensive and often suffers from low temporal resolution. As bodies of water around the world are changing rapidly due to global warming, it is more important than ever to model their spatial variation through time. A point-centered regression CNN (PSRCNN) was used in [73] to analyze lake reflectance data to model water transparency. The authors concluded that their model outperformed different band ratios and traditional ML models (KNN, RF, SVM), although at the cost of generalization. The PSRCNN did not make stable predictions due to too little data.

There is currently not enough paired RS imagery and in situ water measurement to meaningfully create robust water quality monitoring applications. The generation of a synthetic dataset of atmospheric reflectances and its suitability for water quality monitoring were investigated in [76]. The synthetic dataset is physics-based and attempts to capture the natural variability in inland water reflectances and chlorophyll-a concentrations. An ANN outperforms several traditional ML models (KNN, RF, XGBoost) in predicting actual water quality parameter values when trained on the synthetic dataset, although only the ANN is validated against unseen data. Still, synthetic data generation is a promising research direction for water body and water quality detection. Without RS imagery, many water quality monitoring programs will suffer from lack of spatial coverage due to labor, time, and cost constraints. Yet while RS is a useful tool for monitoring water quality parameters, it has not been meaningfully integrated into operational water quality monitoring programs. Existing water quality time series data were used in [75] and assessed the effectiveness of multiple RS data platforms and ML models in estimating various water quality parameters. The authors showed that some sensors are poorly correlated with water quality parameters, while others are more suitable for water quality monitoring tasks. They concluded that more research needs to be carried out for assessing the suitability of paired RS imagery and in situ field data.

Current water quality monitoring systems are labor-, time-, and cost-intensive to operate. IoT sensors can monitor water quality parameters in near real time, allowing for much more data to be recorded with much higher temporal resolution. A wireless sensor network made up in part of IoT sensors was used in [61], and used an MLP to classify water quality as either good or bad. The authors utilized the MLP predictions to notify water quality managers via SMS if the water quality drops below a certain threshold value. However, because of the cost to deploy and run the network, the authors were not able to include additional water quality parameters from more types of bodies of water other than rivers. Water quality monitoring data collection is expensive and time consuming, and there are usually tradeoffs between spatial and temporal resolution when implementing data collection programs. In addition, several key water quality parameters (pH, turbidity, temperature) can be estimated directly from optical and infrared RS imagery. Randrianianina et al. [64] used RS imagery and DNNs to model water quality parameters directly, after which they extend their analysis to map the distributions of water quality parameters to an entire lake, but they only focused on one lake and did not test their methods on other bodies of water.

As bodies of water are exposed to increased nutrient loads, harmful algal blooms can occur, leading to eutrophication. This process can create dead zones that would kill wildlife and lead to negative economic impacts. Thus, it is important to monitor chlorophyll-a levels in water bodies and predict algal blooms before they happen. Zhao et al. [74] attempted to address this need by comparing DL models to traditional ML and curve-fitting methods to predict chlorophyll-a levels using time series measurements paired with RS imagery. The authors did not have much data as they limited the data collection process to one lake. Thus, the DL models did not perform well. Additionally, the ML models used in this paper needed more data and computing than simpler models in order to perform well.

It is often difficult to monitor inland water bodies for quality because of low signal-to-noise ratios and limitations in resolution. A proximal hyperspectral imager was used in [77] with high spectral and temporal time series data for continuous water quality observations. The authors found that index-based methods of water quality detection were difficult to calibrate as thresholding values are subjective, while ML and DL models performed much better. However, the authors show that their models do not generalize well to other water bodies with different water quality parameter distributions.

Anthropogenic activities have currently threatened largely coastal ecosystems. Coastal ecosystems are complex bodies of water but monitoring them is very important. The performance of an ANN was compared to traditional ML models in [62] for predicting various water quality parameters. In some cases, traditional ML methods outperform the ANN. More importantly, the authors conducted an analysis of relative variable importance to show which sets of input data helped the ML models to learn the most. While the relative variable importance analysis is critically important, the authors only test their method in cloud-free RS imagery, limiting its utility. Additionally, while biophysical and chemical water quality parameters were analyzed, little work was carried out with bio-optical data due to issues with data availability.

While recent advances in RS capabilities for water quality detection are substantial in the literature, few papers have collected and synthesized the resources available to researchers. In a paper reviewing recent trends in RS imagery, cloud computing, and ML methods, [67] used time series data from hundreds of water quality parameters and water samples and combined them with proximal imagery, hyperspectral imagery, and two sets of data from different satellite data platforms. They showed that DNNs outperform many other traditional processing and ML techniques for assessing water quality. The authors conclude that anomaly detection using multisensor data is the most promising method for algal bloom detection. As is sometimes the case in the water body detection and water quality monitoring literature, the authors did not have a third holdout set (necessary for DL projects so that the data is not memorized).

## 4. Challenges and Opportunities

In this section, we first provide a brief summary and discussion of the key themes and overall insights (Section 4.1) derived from reviewing the range of research discussed above. In Section 4.2, we provide and discuss some of the major challenges we identified through our systematic survey. Specifically, those challenges shared in both domains are detailed in Section 4.2.1, those specific only to water body extraction in Section 4.2.2, and those specific to water quality monitoring in Section 4.2.3. Finally, we discuss possible research directions and related opportunities for water body detection and water quality monitoring using RS and AI in Section 4.3.

### 4.1. Summary and Discussion

After introducing the essential terms in AI and RS (Appendix B) and commonly used evaluation metrics in ML and DL for classification, regression, and segmentation tasks (Appendix C), we reviewed recent and influential research for water body detection and water quality monitoring using RS and AI (Section 3). 

While the research investigated in Section 3 has demonstrated the power of using RS and AI to detect water bodies and monitor water quality, very few studies thus far performed integrative research of water body and water quality using the power of RS and AI. In addition, most existing RS and AI-based work on water bodies and water quality repeat the same (or very similar) methods in a different research location or on a different (usually small) dataset. However, real intelligent water resource management applications will require serious development that goes beyond this type of research. Before operational applications can be deployed, AI models (especially DL models) need to be trained on large and representative benchmark datasets with a focus on making models generalizable and interpretable. 

We noticed that most work does not include hardware specifications (e.g., what CPU/GPU the authors used to run their models) and/or processing time. To make models comparable and for the sake of replicability and reproducibility, it is essential to report such information. This is even true for index-based methods and more traditional ML models so that researchers can fully evaluate the trade-offs between runtime, accuracy, and ease of implementation. We hope our review will provide a useful guide to make future research more replicable and reproducible. From our interactive web app (the web app tool URL and its brief demo video link are provided in Appendix A), we also noticed that while most papers have an open access PDF/HTML version of their manuscripts, a sizable portion of manuscripts (16 out of 56 of reviewed articles) do not. We suggest authors provide an open access version (e.g., posting the proofreading version after acceptance to ResearchGate/arXiv) in order to increase the visibility of their research and thus to accelerate the advancement of scientific knowledge.

### 4.2. Identified Major Challenges

Below, we provide the most commonly posed challenges for water body and water quality research in the literature we reviewed. Those challenges shared in both domains are outlined in Section 4.2.1 and those specific to each domain are detailed in Section 4.2.2 and Section 4.2.3, respectively. Here are some specific issues to water body detection and water quality monitoring.

#### 4.2.1. Shared Common Challenges in Both Domains

A summary of the shared common challenges and identified problems in water body extraction and water quality monitoring using RS and AI are provided below.

- Methods for water body detection and water quality monitoring need to be able to work quickly and reliably on large spatial and temporal scales, and yet high-resolution RS imagery is very complex. Index methods rely on subjective threshold values that can change over time and space depending on weather conditions. Shallow ML models are more accurate, but do not work at scale. DL models are complex, require very large datasets to train on, and are very computationally expensive; also, the hyperparameter tuning process is very tedious and difficult.
- It is difficult to know exactly what data to feed to ML and DL models, and it is difficult to know what to make of the output predictions. This often requires integrative expertise and/or interdisciplinary collaboration of RS, hydrology, biology, and CV/ML expertise.
- NNs generally perform the best in water quality and water body detection tasks but are often the least stable models (i.e., they do not generalize well). This is not surprising, as the datasets used in RS problem settings are often not large enough to allow NN models (too many parameters compared with shallow ML models) to overcome overfitting (see Appendix B). Table 4 summarizes the relatively few existing datasets we identified through our systematic review.
- Both domains over-rely on optical RS imagery, and thus clouds and shadows are a persistent problem and heavily skew the results towards working only in cloud-free conditions.

#### 4.2.2. Additional Challenges in Water Body Extraction

The specific challenges and problems identified for water body extraction are summarized below.

- The majority of reviewed research focused on inland bodies of water, where only a few papers discussed applications for coastal waters (not including oceans). Moreover, many papers focus solely on only one type of water body, for example, only on lakes or rivers in a specific area. As a result, water bodies from different landscapes (e.g., inland, coastal tidal flats, urban, wetlands) are difficult to recognize with one unified method (i.e., methods do not generalize). The same applies to water bodies of different colors, especially when distinguishing them from rock, ice, snow, clouds, and shadows.
- There are very few benchmark datasets. In contrast, there are huge volumes of unlabeled data not being fully leveraged.
- CNNs blur output boundaries during the segmentation process.

#### 4.2.3. Additional Challenges in Water Quality Monitoring

The specific challenges and problems identified for water quality monitoring are summarized below.

- Collecting in situ water quality data is very time- and labor-intensive and financially expensive; also, it often does not have adequate temporal or spatial resolution.
- RS imagery and existing corresponding field samples are often not stored together. Allowing water quality researchers to easily retrieve and locate two or more sources of data at the same location is critical, as computational methods require such data to verify their model performance in order to generalize to new water bodies.
- Remote water bodies are difficult to monitor.
- Urbanization, pollution, and drought are having serious effects on the economy, wildlife, and human health as they deteriorate water quality.
- Ecosystems are complex and their nutrient and pollution budgets are not well understood.
- Some studies do not use a training, validation, and testing set for DL projects (all three are necessary) or do not use nearly enough data to achieve good results with DL models.

### 4.3. Research Directions and Opportunities

Here, we provide five research directions, each along with its promising opportunities, from our investigation and based on the posed challenges discussed in Section 4.2 above.

#### 4.3.1. Urgent Need of Large and Comprehensive Benchmark Datasets

Large representative, balanced, and open-access benchmark datasets are critical for any domain to let AI meaningfully shine [84,85,86]. In computer science, especially for its branches CV and DL, there are very comprehensive, large, and open-source databases (e.g., ImageNet [87] for image classification tasks, and Microsoft COCO [88] for object detection and segmentation tasks). The availability of big and open-source image repositories has dramatically boosted recent advances in novel and robust algorithms in DL and CV, as computer science researchers do not need to worry about collecting datasets. Instead, they can focus on developing new algorithms and/or methods.

In our systematic review, we identified an urgent need for more curated, labeled datasets for intelligent water body extraction and water quality monitoring. We found some of the few available open-source datasets with water body boundary labels through our literature review, but also sought out additional datasets. We identified datasets that were not used in our literature review but contain water body labels, or datasets that were used for water body detection or water quality monitoring that did not use ML/DL/CV but would be useful for benchmarking tasks. Our search results are summarized in Table 4 above. Below, we list a few opportunities in this direction.

(1) *More public data and code: currently, most authors do not share their code and/or datasets.* See the two quoted pieces below from [25]: (a) “Lack of deep learning-ready datasets within the water field […] The main problem caused by this absence of many datasets is that the research community does not build upon previous work in terms of constructing better neural network architectures and moving the state of art to the next iteration […]”; (b) “[…] many papers are published that achieve the same task with almost identical methods but different data.”. Part of this issue is a replication crisis in the water body detection and water quality monitoring literature, but it stems more broadly from the lack of public codebases and datasets.

(2) Some promising ways to generate large datasets of good quality

- AI/ML/DL models need large datasets with good quality to guarantee meaningful (unbiased and generalize well) good to great performance, thus work on obtaining large but better subsets of data. Quality > quantity is critical and in urgent demand. See one piece of such evidence reported in [44], “[…] site-specific models improved as more training data was sampled from the area to be mapped, with the best models created from the maximum training datasets studied: […] However, performance did not improve consistently for sites at the intermediate training data thresholds. This outcome exemplifies that model improvement is an issue of not only increasing the quantity of training data, but also the quality”.
- Generating synthetic data as in [76] (detailed in the second paragraph in Section 3.3.5).
- Downloading RS images from Google Earth Engine (GEE) and annotating accordingly, or, even better, developing user-friendly interactive interfaces with GEE as a backend to directly allow researchers (or even citizen science volunteers) to contribute to the annotation of RS imagery available on GEE. To our knowledge, no RS datasets for water body detection and water quality monitoring are downloaded from GEE and then annotated, let alone interfaces for directly annotating RS imagery on GEE.
- Obtaining RS imagery from Google Earth (GE) manually or with the help of code scripts, then annotating accordingly (see [34,42,49] for examples). For instance, the following two datasets generated and used in [34,49] are both from GE, but are not shared publicly.
  ○ “The first dataset was collected from the Google Earth service using the BIGEMAP software (http://www.bigemap.com, accessed on 15 December 2021). We named it as the GE-Water dataset. The GE-Water dataset contains 9000 images covering water bodies of different types, varying shapes and sizes, and diverse surface and environmental conditions all around the world. These images were mainly captured by the QuickBird and Land remote-sensing satellite (Landsat) 7 systems.” [49].
  ○ “We constructed a new water-body data set of visible spectrum Google Earth images, which consists of RGB pan-sharpened images of a 0.5 m resolution, no infrared bands, or digital elevation models are provided. All images are taken from Suzhou and Wuhan, China, with rural areas as primary. The positive annotations include lakes, reservoirs, rivers, ponds, paddies, and ditches, while all other pixels are treated as negative. These images were then divided into patches with no overlap, which provided us with 9000 images […]” [34].

#### 4.3.2. Generalization

It is important to be able to obtain a good accuracy score when training an ML/DL model, but perhaps more important is that model’s ability to generalize to unseen data. The ultimate goal of ML/DL is to develop predictive models through finding statistical patterns in a training set which then generalize well to new, previously unseen data outside the training set [89]. Ideally, this is achieved by training on large and representative datasets that capture nearly all variations in the data actual distribution of values [86,89]. A model’s ability to generalize is critical to the success of a model. An ML/DL model with good generalization capability will have the best trade-off between underfitting and overfitting so that a trained model obtains the best performance (See “Generalization, overfitting, underfitting and regularization” entry in Appendix B for details). Below, we outline a few ways to make AI systems more generalizable for water body detection and water quality monitoring tasks.

(1) Create robust AI methods for tiny water body detection. Depending on resolution, tiny water bodies such as ponds or small lakes in desert cities are difficult to identify yet may play a more critical role than we think.

(2) Develop NN architectures and comprehensive datasets (see Section 4.3.1) that are able to recognize water bodies not just from

- One type of body of water (e.g., ponds, lakes, rivers);
- One color (e.g., different levels of sediment, aquatic vegetation and algae, nutrients, pollutants);
- One size: Water bodies present in RS imagery come with different sizes (large and small water bodies) and various shapes. Many studies reported that it is not an easy task to correctly classify small water bodies and/or water bodies with different shapes.
- One environment setting (e.g., desert, urban, inland, coastal).

(3) Utilize data from multiple sources to train ML/DL models. From our comprehensive investigation, most of the current AI methods are only able to deal with water quality and/or water body detection data from one specific type of RS imagery. This should be improved and indicates a promising new research direction. Specifically, it will be important to focus on using data from multiple data platforms or resolutions, from varying weather conditions, and regions which have different ecosystem and terrain types. We humans can recognize water bodies in different RS imagery with different weather conditions. We expect that machines should be able to mimic humans to perform this task well if we have robust AI algorithms and comprehensive datasets. See some example research below:

- Extraction of water bodies at multiple resolutions and scales using CNNs [49,53];
- Evaluation of CNN performance on multisensor data from multiple RS platforms [52];
- Integration of data from multiple sources (e.g., SAR, UAV, smaller sensors, water quality time series);
- Data fusion of Landsat-8 and Sentinel-2 RS imagery for water quality estimation [67]. “Virtual constellation” learning introduced in [67] could be a future direction for both water body detection and water quality estimation. A virtual constellation is constructed by using multiple RS platforms to “shorten” the revisit time and improve the spatial coverage of individual satellites. This entails fusing data sources from separate RS platforms with potentially different resolutions.

(4) Propose new frameworks for improving generalizability. Generalization is one of the fundamental unsolved problems in DL. The goal of a generalization theory in supervised learning is to understand when and why trained ML/DL models have small test errors [90]. The recently proposed deep bootstrap framework [90] provides a new lens for understanding generalization in DL. This new framework has the potential to advance our understanding of water domain research empowered by RS and AI by highlighting important design choices when processing RS imagery with DL.

#### 4.3.3. Addressing Interpretability

DL has achieved significant advances with great performance in many tasks in a variety of domains, including some water domain tasks (detailed in Section 3). In the literature we reviewed for this paper, DL models have produced results comparable to, and in some scenarios even superior to, human experts. Improving predictive accuracy is important; however, improving the interpretability of ML/DL models is more important, especially through visualization techniques of ML/DL model output for later analysis by humans [18]. Interpretability is one of the primary weaknesses of DL techniques and raises wide concerns and attention in DL [91]. Due to the overparameterized and black-box nature of DL models, it is often difficult to understand the prediction results of DL models [92,93]. Understanding and explaining their black-box behaviors remains challenging due to their hierarchical, nonlinear nature. The lack of interpretability raises major concerns across several domains; for example, in high-stakes prediction applications, such as autonomous driving, healthcare, and financial services [94], the trust of DL models is critical. While many interpretation tools (e.g., image perturbation and occlusion [95], visualizing NN activation weights and class activation mapping [96,97] or attention mechanisms [98,99], feature inversion [100], local interpretable model-agnostic explanations or “LIME” [101]) have been proposed to interpret how DL models make decisions, either from a scientific perspective or a social angle, explaining the behaviors of DL models is still in progress [92]. For water domains, we list some specific potential opportunities in terms of interpretability we identified below.

- More ablation studies are needed (see Appendix B for an introduction) to investigate the role of each DL component in terms of model performance contribution and ultimately which component(s) control the model performance.
- Exploring the output of hidden layers to obtain some information to help investigate whether the model works as expected.
- Hybrid models for analyzing NN output and improving an NN’s decision-making process through post-processing, for example, CNN–LR hybrids [32], CNN–CRF hybrids [36,38], CNN–SVM hybrids [39], RNN–DS hybrids [63], and CNN-LSTM hybrids [69].
- More research needs to be carried out on analyzing the importance of input data to output predictions. See examples in [62,75], each detailed below.
  ○ The authors in [62] systematically analyzed relative variable importance to show which sets of input data contributed to the ML models’ performance. See the quoted text below: “Relative variable importance was also conducted to investigate the consistency between in situ reflectance data and satellite data, and results show that both datasets are similar. The red band (wavelength ≈ 0.665 µm) and the product of red and green band (wavelength ≈ 0.560 µm) were influential inputs in both reflectance data sets for estimating SS and turbidity, and the ratio between red and blue band (wavelength ≈ 0.490 µm) as well as the ratio between infrared (wavelength ≈ 0.865 µm) and blue band and green band proved to be more useful for the estimation of Chl-a concentration, due to their sensitivity to high turbidity in the coastal waters”.
  ○ The authors in [75] utilized existing water quality time series data and assessed the effectiveness of multiple RS data platforms and ML models in estimating various water quality parameters. One of their interesting findings is that some sensors are poorly correlated with water quality parameters, while others are more suitable for water quality monitoring tasks. They suggested that more research needs to be carried out for assessing the suitability of paired RS imagery and in situ field data. See the quoted text below: “[…] assess the efficacy of available sensors to complement the often limited field measurements from such programs and build models that support monitoring tasks […] We observed that OLCI Level-2 Products are poorly correlated with the RNMCA data and it is not feasible to rely only on them to support monitoring operations. However, OLCI atmospherically corrected data is useful to develop accurate models using an ELM, particularly for Turbidity (R^2^ = 0.7).” (RNMCA is the acronym for the Mexican national water quality monitoring system).
- Water quality monitoring will benefit from more research exploring how well a certain ML/DL model contributes to which water quality parameter(s). See an example in [67], where the authors investigated how well DNNs could predict certain water quality parameters.
- Physics-constrained or process-based ML/DL predictions as demonstrated in [68,69].
- The need for automatic and visually-based model evaluation metrics that are better than current visual assessment as an evaluation metric. For example, automatic assessment of how DL methods are performing in large and complex RS imagery (e.g., specifically, Bayesian DL, and Gaussian DL/ML for uncertainty measurement and visualization).

#### 4.3.4. Ease of Use

As emphasized in [13,14], one of the major current challenges for water resource management is the integration of water quality data and indices from multiple sources into usable and meaningful insights for actionable management decisions. Geovisualization, also known as geographic visualization, uses the visual representations of geospatial data and the use of cartographic techniques to facilitate thinking, understanding, knowledge construction, and decision support about human and physical environments at geographic scales of measurement [102,103]. Geovisualization is widely utilized in different domains (e.g., public health [104], crisis management [105,106], environmental analysis [107,108,109], and climate change strategies [110]) for the exploration and analysis of spatiotemporal data. To the best of our knowledge, very little research has leveraged geovisualization in this way for water resources management. The only piece of work similar to this we noticed is in [111], where a web interface powered by GEE allows their expert system, combined with visual analytics, to be run on any Landsat 5, 7, or 8 imagery to draw boundaries for water bodies. Geovisualization through interactive web applications provides a promising solution to the posed challenge of integrating water quality data and indices from multiple sources [112,113,114,115]. We provide a few suggested research opportunities in this direction below.

- Simply applying (or with minor modifications) existing AI/ML/CV/DL algorithms/methods to RS big data imagery-based problems is still very far away from producing real-world applications that meet water management professionals’ and policymakers’ needs. As echoed in [13], “[…] realizing the full application potential of emerging technologies requires solutions for merging various measurement techniques and platforms into useful information for actionable management decisions, requiring effective communication between data providers and water resource managers” [116]. Much more multidisciplinary and integrative collaboration in terms of depth and breadth are in high demand. Those scholars and practitioners who have an interdisciplinary background will play a major role in this in-depth and in-breadth integration. For example, researchers who have expertise in RS but also know how to utilize AI, through collaboration with domain expertise such as water resources management officers, will significantly advance this research direction. Intuitive interactive web apps that are powered by both geovisualization and AI/ML/DL/CV will definitely make interdisciplinary collaboration much more seamless and thus easier.
  ○ Interactive web portal empowered by geovisualization for integration of various water quality data sources. As noted in [117], it is natural and intuitive in many studies to use “space” as the organizing paradigm.
  ○ More smart and responsive water management systems through the development of interactive web apps/libraries that integrate ML/DL backends and intuitive, user-friendly front ends are needed. Such systems would allow collaboration between technical experts and domain experts, including stakeholders, and even community volunteers, from anywhere at any time.
  ○ This requires very close collaboration and thus very integrative research from researchers in many domains (e.g., computer science, cognitive science, informatics, RS, and water-related sub-domains). We reinforce that geovisualization will be the ideal tool to make the collaboration smooth, productive, and insightful.
  ○ There is one recent work [118] that takes a small step in this direction, but much more work and efforts are in demand.
- Resource hubs for standardized AI/ML/DL/CV models and easy-to-follow and understandable tutorials for how to use them are needed.
- More data “matchups” as demonstrated in [82,83]. When more in situ measurements come in, they should be matched up and stored with satellite data for easy calibration studies.

#### 4.3.5. Shifting Focus

From our investigation, it is clear that with enough annotated data and allocated computing, DL models are more accurate than traditional ML models, which are in turn more accurate than index-based methods for water body detection and water quality monitoring tasks. Increasing the accuracy of models by fractions of a percent should be given much less focus and attention moving forward. Water body detection methods are unlikely to improve upon the high rates of accuracy already reported in the literature without very high-resolution, very large, labeled datasets or the use of UAVs to detect small water bodies. Instead, we suggest that future research should focus more on reducing model parameters and making model training less computationally expensive in terms of time (e.g., designing neural networks to use constant memory at inference time [40], or by using TL [37,59]). Below, we outline some additional potential research directions we identified through our systematic review.

- As noted in Section 4.3.1, the lack of large benchmark datasets is a bottleneck in water body detection and water quality monitoring research utilizing RS imagery and AI. The dominant methods in both water domains are supervised learning, which often requires very large, labeled datasets to train on, thus, there is a clear, urgent need for semi-supervised and unsupervised learning methods [15].
  ○ Unsupervised learning methods are able to learn from big sets of *unlabeled* data, as demonstrated in [29,46].
  ○ Semi-supervised learning methods are able to learn from limited good-quality labeled samples. DL models do not require feature engineering, and they are also much better at discovering intricate patterns hidden in big data. However, pure supervised DL is impractical in some situations, such as those for which the labeling tasks require domain knowledge from experts. Very few domain experts have the time and are willing to label very large sets of RS images [84]. An active learning-enabled DL approach that uses a visualization interface and methods to iteratively collect modest amounts of input from domain experts and uses that input to refine the DL classifiers [84] provides a promising direction to produce well-performing DL models with limited good-quality datasets.
- From our systematic review, we can easily see that current work on water body extraction and water quality monitoring using AI and RS are, in general, carried out separately. We call for a closer integration of water body detection and water quality monitoring research and more attention focusing on handling massive datasets that may include information in a variety of formats, of varying quality, and from diverse sources. This integration is critical as it will provide the essential foundation for developing real, intelligent water monitoring systems using RS and AI capable of producing insights used for actionable decision making.
- GEE + AI: as noted in [18], GEE is a good solution to address computational costs and overcome technical challenges of processing RS big data. However, online DL functionality is still not supported on GEE. To the best of our knowledge, the only piece of research integration of the Google AI platform with GEE is performed in [119]; however, as the authors reported, “data migration and computational demands are among the main present constraints in deploying these technologies in an operational setting”. Thus, the ideal solution is to develop DL models directly on the GEE platform.
- Most current ML/DL-based RS research focuses on borrowing or slightly improving ML/DL/CV models from computer science [79,120]. Compared with natural scene images, RS data are multiresolution, multitemporal, multispectral, multiview, and multitarget [15]. Slight modifications of ML/DL/CV models simply cannot cope with the special challenges posed in RS big data. New ML/DL models specialized for RS big data are thus urgently needed [15,18]. We hope our review will draw the attention of researchers who have a multidisciplinary background to this issue. Looking deep into the mechanisms of RS and land surface processes, studying the characteristics of RS imagery would guide the design of specialized ML/DL models for RS big data and thus further improve RS applications using AI in breadth and depth [15].

## 5. Conclusions

Building intelligent and synoptic water monitoring systems requires automation of water body extent detection using RS imagery, from which volume can be computed, and also automation of their corresponding water quality, eventually linking the two to allow synoptic water quality monitoring. Yet, to date, water body detection and water quality monitoring research has been historically separate. Our systematic investigation indicates the following trends: deep learning is much more commonly used in water body detection, the dominant data source of which is RS imagery, whereas water quality literature often involves other types of data sources (e.g., in situ sensors, smaller RS devices that are not satellites). The trends relate to the scale of projects in the two domains: water body extraction is usually undertaken across large spatial scales, whereas the water quality monitoring literature is still only focused on smaller, often individual, bodies of water. This points to one of the future research directions in the water quality literature that we touch on above in Section 4.3; that is, we need to scale up water quality estimation using RS imagery through matching it with ground-truth water quality measurements. 

Overall, based on the systematic review above, we contend that RS integrated with AI/ML/DL/CV methods, along with geovisualization, have great potential to provide smart and intelligent support for water resources monitoring and management. Thus, this integration has considerable potential to address major scientific and societal challenges, such as climate change and natural hazards risk management.

## Figures and Tables

**Table 1:** Keywords used for article search.

Keyword Category	Search Strategy
General keywords ^1^	“remote sensing” OR “satellite data” OR “UAV” AND “computer vision” OR “machine learning” OR “deep learning” OR “neural networks” OR “AI”
Water body	“water body” AND “detection” OR “extraction”
Water quality	“water quality” AND “sensing” OR “monitoring”

^1^ A list of general keywords were combined with either the category of water body or water quality, respectively, to perform our search.

**Table 2:** Studies targeting water body detection from RS imagery using AI (note that it is ordered chronologically to show trends in data type and model usage; see the Abbreviations for a list of the acronyms).

Reference	Method	Model Comparison	RS Data Type	Evaluation Metrics
Li et al. (2011) [28]	DNN	NDWI	Landsat TM	coherence
Yang et al. (2015) [29]	AE	DNN, SVM	Landsat ETM+	accuracy
Huang et al. (2015) [30]	ELM	DT, LORSAL, RF, SVM, TB	GeoEye-1, WorldView-2	Kappa, F-score
Isikdogan et al. (2017) [31]	CNN	MDWI, MLP	Landsat	F1-score, CE, OE, precision, recall
Yu et al. (2017) [32]	CNN–LR hybrid	ANN, CNN, SVM	Landsat ETM+	accuracy
Jiang et al. (2018) [10]	MLP	MLC, NDWI	Landsat-8 OLI	Kappa, OA
Chen et al. (2018) [33]	CNN	CNN, NDWI, SVM	GaoFen-2, Zi Yuan-3	ECE, EOA, EOE, OA, PA, UA
Miao et al. (2018) [34]	CNN	DNN	Google Earth imagery	OA
Acharya et al. (2019) [35]	SVM	ANN, DT, GMB, NB, NDWI, RF, RPART	Landsat-8 OLI	Kappa, OA
Feng et al. (2019) [36]	CNN–CRF hybrid	CNN, CV-method	GaoFen-2, WorldView-2	Kappa, PCC, precision
Li et al. (2019) [37]	CNN	CNN, NDWI, SVM	GaoFen-2	F1-score
Li et al. (2019) [38]	CNN–CRF hybrid	CNN, NDWI	GaoFen-1	IoU, pixel accuracy, recall
Meng et al. (2019) [39]	CNN–SVM hybrid	CNN, SVM	GaoFen-2	accuracy, MA, UA
Isikdogan et al. (2020) [40]	CNN	CNN, MLP, MNDWI	Landsat-8	F1-score, precision, recall
Song et al. (2020) [41]	CNN	CART, KNN, RF, SVM	GaoFen-2, WorldView-3	IoU, precision, recall
Yang et al. (2020) [42]	CNN	CNN	GaoFen-2	IoU
Wang et al. (2020) [43]	CNN	CNN, NDWI	GaoFen-1	F1-score, mIoU, precision, recall
O’Neil et al. (2020) [44]	CNN	DEM, NDVI, RF	LiDAR DEMs, NAIP	precision, recall
Chen et al. (2020) [45]	CNN	NDWI, SVM	GaoFen-1, GaoFen-2, Zi Yuan-3	BOA, Kappa, OA
Dang and Li (2021) [46]	CNN	CNN	GaoFen-2, GID	mIoU, FWIoU, OA
Yuan et al. (2021) [47]	CNN	CNN, MNDWI, NDMI, NDWI	Sentinel-2	accuracy, mIoU
Tambe et al. (2021) [48]	CNN	CNN	Landsat-8 OLI	CA, F1-score, GA, IoU, precision, recall
Yu et al. (2021) [49]	CNN	CNN	GaoFen-2, Landsat-7	F1-score, OA, precision, recall
Li et al. (2021) [50]	CNN	CNN, CV-method, SVM	UAV	Kappa, F-score, OA, precision
Zhang et al. (2021) [51]	CNN	CNN, MLC, NDWI, SVM	GaoFen-2	IoU, Kappa, pixel accuracy
Li et al. (2021) [52]	CNN	CNN, NDWI, SVM	GaoFen-2, GaoFen-6, Sentinel-2, Zi Yuan-3	F1-score, IoU, OA
Su et al. (2021) [53]	CNN	CNN	Landsat-8, Sentinel-2A	IoU, pixel accuracy, recall
Ovakoglou et al. (2021) [54]	KMeans	fuzzy-rules classification, Haralick’s textural features of dissimilarity, Otsu valley-emphasis	Sentinel-1	Kappa, OA, precision, recall

**Table 3:** Studies targeting water quality monitoring from RS imagery using AI (where “/” means none. Note that it is ordered chronologically to show trends in data type and model usage) (See the Abbreviations for a full list of the acronyms).

Reference	Method	Model Comparison	RS Data Type	Evaluation Metrics
Chebud et al. (2012) [55]	DNN	/	Landsat TM	RMSE, R^2^
Wang et al. (2017) [56]	SVR	index methods	spectroradiometer, water samples	RMSE, RPD, R^2^
Lee and Lee (2018) [57]	LSTM	DNN, RNN	water quality time series	RMSE
Wang et al. (2019) [58]	LSTM	/	water quality time series	accuracy, cross-correlation
Pu et al. (2019) [59]	CNN	RF, SVM	Landsat-8	accuracy
Liu et al. (2019) [60]	LSTM	ARIMA, SVM	IoT data	MSE
Chowdury et al. (2019) [61]	MLP	/	IoT data	threshold value
Hafeez et al. (2019) [62]	DNN	CB, RF, SVR	Landsat	accuracy, relative variable importance
Li et al. (2019) [63]	RNN–DS hybrid	GRU, LSTM, SRN, SVR	water quality time series	MAE, MAPE, RMSE
Randrianiaina et al. (2019) [64]	DNN	/	Landsat-8	RMSE, R^2^
Yu et al. (2020) [65]	LSTM	/	water quality time series	MAE, RMSE
Zou et al. (2020) [66]	LSTM	DNN, GRU, LSTM	meteorological time series, water quality time series	MAE
Peterson et al. (2020) [67]	ELR	MLR, SVR	Landsat-8, Sentinel-2	MAPE, RMSE, R^2^
Hanson et al. (2020) [68]	LSTM	/	water quality time series	auto-correlation, MK statistics, RMSE
Barzegar et al. (2020) [69]	CNN–LSTM hybrid	CNN, LSTM	water quality data from multiprobe sensor	MAE, NSEC, Percentage of Bias, RMSE, Wilmott’s index
Aldhyani et al. (2020) [70]	LSTM	ANN, DNN, KNN, NB, SVM	water quality time series	accuracy, F-score, MSE, precision, R, sensitivity, specificity
Li et al. (2021) [71]	RF	SVM	Sentinel-2 MSI	RMSE, RPD, R^2^, Z-score
Sharma et al. (2021) [72]	CNN	CNN	UAV camera	precision, recall
Cui et al. (2021) [73]	CNN	KNN, index method, RF, SVM	Landsat-8, Sentinel-2	RPD, RMSE, R^2^
Zhao et al. (2021) [74]	DNN	RBFNN	Landsat-8, water quality time series	MAE, MSE, R^2^
Arias-Rodriguez et al. (2021) [75]	ELM	LR, SVR	Landsat-8, Sentinel-2 MSI, Sentinel-3 OLI	MAE, MSE, RMSE, R^2^
Kravitz et al. (2021) [76]	DNN	KNN, RF, XGBoost	Landsat 8 OLI, Sentinel-2 MSI	MAPE, RMSE, RMSLE ^1^
Sun et al. (2021) [77]	DNN	GPR, RF	proximal hyperspectral imager, water samples	accuracy, MRE, RMSE, R^2^

^1^ The authors use the abbreviation RMSELE for RMSLE in their paper (this might be a typographical error).

**Table 4:** Existing datasets for waterbody extraction and water quality monitoring.

Datasets	Source	Category	Link to the Dataset	Notes
DeepWaterMap v2	[40]	Water body	https://github.com/isikdogan/deepwatermap, accessed on 15 December 2021	>1 TB of Landsat-7 imagery paired with Global Inland Water dataset labels.
2020 GaoFen Challenge	/	Water body	https://github.com/AICyberTeam/2020Gaofen, accessed on 15 December 2021	Dataset containing both 2500 optical and 1200 SAR satellite images with pixel level labels for water body segmentation.
GID-15	[79]	Water body	https://captain-whu.github.io/GID15/, accessed on 15 December 2021	150 pixel-level annotated GaoFen-2 images for semantic segmentation tasks.
LandCover.ai	[80]	Water body	https://landcover.ai/, accessed on 15 December 2021	A dataset from 2015–2018 of 10,674 annotated tiles of RGB imagery with labeled water bodies.
SEN12MS	[81]	Water body	https://mediatum.ub.tum.de/1474000, accessed on 15 December 2021	A curated dataset of 180, 662 georeferenced multispectral Sentinel-1 and -2 imagery with MODIS land cover labels.
AquaSat	[82]	Water quality	https://github.com/GlobalHydrologyLab/AquaSat, accessed on 15 December 2021	600,000 data matchups between satellite imagery and water quality measurements from 1984–2019.
Forel–Ule Index	[83]	Water quality	https://doi.org/10.6084/m9.figshare.13014299, accessed on 15 December 2021	151 data matchups between satellite imagery and water quality measurements from 2000–2018.

**Figure A1:**
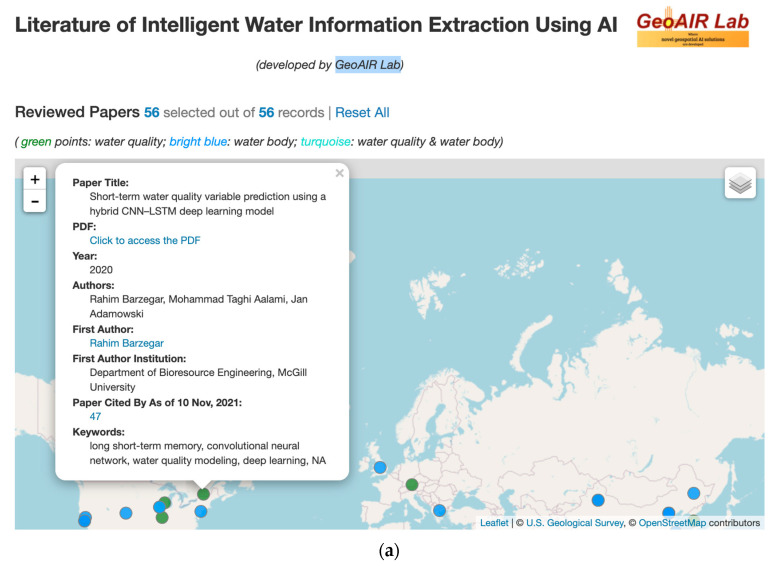

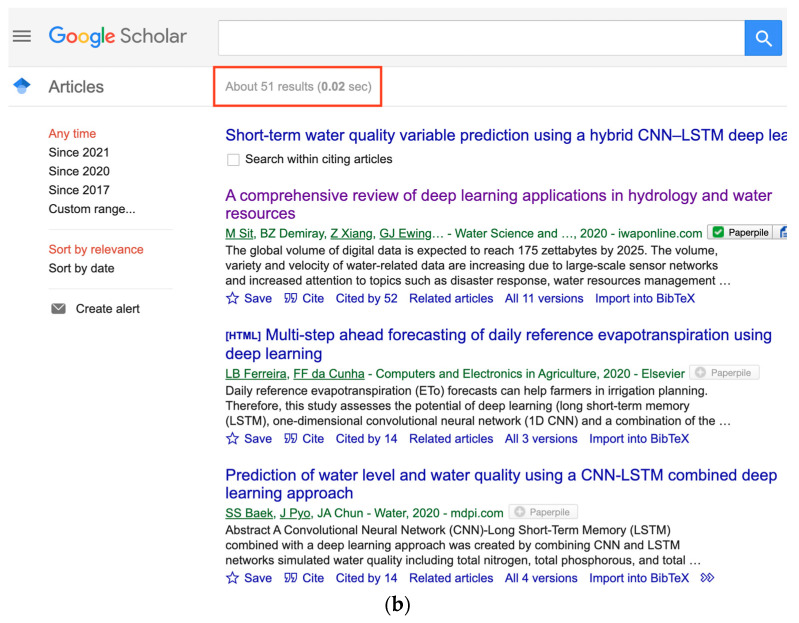
Our highly interactive web app (accessible publicly at: https://geoair-lab.github.io/WaterFeatureAI-WebApp/index.html, accessed on 5 December 2021) provides the track of scholars and publications with just a few clicks. See an example on the pop-up. Our readers can access (1) a direct link to the PDF file of the paper (note that if there is no free, publicly available version of the paper, we link directly to the journal page of the paper so our readers can obtain the paper if their institution purchases the journal database), (2) the scholar profile (Google Scholar/ResearchGate URL) of the first author, and (3) “Cited by” Google Scholar page. (**a**) Water body and quality AI literature map pop-up. (**b**) “Cited by” Google Scholar page corresponding to the paper shown in (**a**).

**Figure A2:**
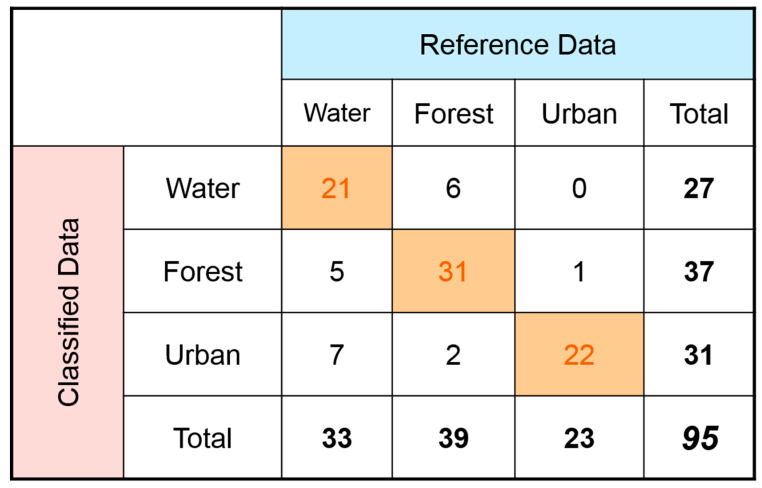
Example confusion matrix. The classified data indicate the ML/DL model predicted results and the reference data refer to the actual manually annotated data (image source: [161]).

## Data Availability

Not applicable.

## Abbreviations

The following abbreviations (in alphabetical order) are used in this manuscript:

AE	Autoencoder
AI	Artificial Intelligence
ANN	Artificial Neural Network
ARIMA	Autoregressive Integrated Moving Average
BOA	Boundary Overall Accuracy
CA	Class Accuracy
CART	Classification and Regression Trees
CB	Cubist Regression
CE	Commission Error
CNN	Convolutional Neural Network
COCO	Common Objects in Context
CPU	Central Processing Unit
CRF	Conditional Random Field
CV	Computer Vision
DL	Deep Learning
DNN	Dense Neural Network
DS	Dempster–Shafer Evidence Theory
DT	Decision Tree
DEM	Digital Elevation Model
ECE	Edge Commission Error
ELM	Extreme Learning Machine
ELR	Extreme Learning Regression
ESA	European Space Agency
EOE	Edge Omission Error
EOA	Edge Overall Accuracy
FN	False Negative
FP	False Positive
FWIoU	Frequency Weighted Intersection over Union
GA	Global Accuracy
GAN	Generative Adversarial Network
GBM	Gradient Boosted Machine
GE	Google Earth
GEE	Google Earth Engine
GPR	Gaussian Process Regression
GPU	Graphics Processing Unit
GRU	Gated Recurrent Unit
IoT	Internet of Things
IoU	Intersection over Union
Kappa	Kappa Coefficient
KNN	K-Nearest Neighbors Classifier
LORSAL	Logistic Regression via Variable Splitting and Augmented Lagrangian
LSTM	Long Short-Term Memory
MA	Mapping Accuracy
MAE	Mean Absolute Error
MAPE	Mean Absolute Percentage Error
mIoU	Mean Intersection over Union
MK	Mann–Kendall
ML	Machine Learning
MLC	Maximum-Likelihood Classifier
MLP	Multilayer Perceptron
MLR	Multiple Linear Regression
MNDWI	Modified Normalized Difference Water Index
MPC	Microsoft Planetary Computer
MRE	Mean Relative Error
MSE	Mean Squared Error
MSI	Morphological Shadow Index
NB	Naive Bayes Classifier
NDMI	Normalized Difference Moisture Index
NDVI	Normalized Difference Vegetation Index
NDWI	Normalized Difference Water Index
NIR	Near-Infrared
NN	Neural Network
NSEC	Nash–Sutcliffe Efficiency Coefficient
OA	Overall Accuracy
OE	Omission Error
PA	Producer’s Accuracy
PCC	Percent Classified Correctly
RBFNN	Radial Basis Function Neural Network
R-CNN	Region Based Convolutional Neural Network
RF	Random Forests
RMSE	Root Mean Squared Error
RMSLE	Root Mean Squared Log Error (referred to in Table 3 as RMSELE by the authors)
RNN	Recurrent Neural Network
RPART	Recursive Partitioning And Regression Trees
RPD	Relative Percent Difference
RS	Remote Sensing
SAR	Synthetic Aperture Radar
SRN	Simple Recurrent Network (same abbreviation given for Elman Neural Network)
SOTA	State-of-the-Art
SVM	Support Vector Machine
SVR	Support Vector Regression
SWIR	Short Wave Infrared
TB	Tree Bagger
TL	Transfer Learning
TN	True Negative
TP	True Positive
VHR	Very High Resolution
UA	User’s Accuracy
UAV	Unmanned Aerial Vehicle

## Appendix A. The Accompanying Interactive Web App Tool for the Literature of Intelligent Water Information Extraction Using AI

In Section 1.1, we provided a brief map and graphic summary of the papers covered in this review. To allow readers to obtain more useful and dynamic information and insights from the papers reviewed, we have developed an interactive web app. Through the web app, readers can keep track of the major researchers and access an up-to-date list of publications in the reviewed topics. Updated publications are accessible through (1) a researcher’s public academic profile on Google Scholar or ResearchGate (see Figure A1a for an example), and (2) a continuously updated citations count of the papers that we reviewed in this paper (see Figure A1b for an example: the cited by as of 10 November 2021 is 47, which is when we first entered the data in our data file when we reviewed the paper, and then before this paper submission, when we clicked on the cited by URL, the page shows that the up-to-date citation number is 49). The web app can be accessed publicly, *free of charge* at

- Web app tool: https://geoair-lab.github.io/WaterFeatureAI-WebApp/index.html, accessed on 28 February 2022.
- Brief web app demo video (about 6 min duration): the video link is accessible at the web app page.

## Appendix B. Essential AI/ML/DL/CV Terms

In this appendix, we provide brief definitions to some essential terms (ordered alphabetically) in ML/DL/RS in our review. For readability, we group some related concepts together.

**Ablation Studies:** In AI, particularly in ML and DL, ablation is the removal of a component of an AI system. Ablation studies are crucial for AI, especially for DL research. An ablation study investigates the performance of an AI system by removing certain components to understand the contribution of the component to the overall system. The term is analogous to ablation in biology (removal of components of an organism). Note that ablation studies require that the systems exhibit *graceful degradation* (i.e., they continue to function even when certain components are missing or degraded). The motivation was that, while individual components are engineered, the contribution of an individual component to the overall system performance is not clear; removing components allows this analysis. Simpler is better: if we can obtain the same performance with two models, we prefer the simpler one.

**Convolution, kernel (i.e., filter), and feature map** [121,122,123]:

Convolutional layers are the major building blocks in CNNs. A convolution is the simple application of a filter (i.e., kernel) to an input that results in an activation. Repeated application of the same filter to an input results in a map of activations called a feature map, indicating the locations and strength of a detected feature in an input (e.g., an image).

Convolution: Convolution is one of the most important operations in signal and image processing. Convolution is a mathematical operation to merge two sets of information. Convolution provides a way of multiplying together two arrays of numbers, generally of different sizes, but of the same dimensionality, to produce a third array of numbers of the same dimensionality. This can be used in image processing to implement operators whose output pixel values are simple linear combinations of certain input pixel values.

A convolutional filter (i.e., kernel) is a weight matrix (vector for one-dimensional and *cube* for three-dimensional data) which operates through a sliding window on input data. The convolution is performed by determining the value of a central pixel through adding the weighted pixel values of all its neighbors together. Specifically, it is carried out by sliding the kernel over the input image, generally starting at the top left corner, so as to move the kernel through all the positions where the kernel fits entirely within the boundaries of the input image. Each kernel position corresponds to a single output pixel, the value of which is calculated by multiplying together the kernel value and the underlying image pixel value for each of the cells in the kernel, and then adding all these numbers together. The output is a new modified filtered image. Convolution is a general purpose filter effect for images. Depending on the kernel structure, the operation enhances some features of the input data (e.g., blurring, sharpening, and edge detection).

In the context of a CNN, a convolution is a linear operation that involves the multiplication of a set of weights with the input. Given that the technique was designed for two-dimensional input, the multiplication is performed between an array of input data and a two-dimensional array of weights (i.e., a filter or a kernel). Technically, note that in CNNs, although it is referred to as a “convolution” operation, it is actually a “cross-correlation”. That is, in CNNs, the filter is not flipped as is required in typical image convolutions; except for this flip, both operations are identical.

Kernel (i.e., filter): A kernel is a small matrix used in image convolution, which slides over the input image from left to right and top to bottom. Differently sized kernels, which contain different patterns of numbers, produce different results through convolution operation. The size of a kernel is arbitrary, but 3 × 3 or 5 × 5 is often used. Think of a filter similar to a membrane that allows only the desired qualities of the input to pass through it.

Feature map: The feature maps of a CNN capture the application result of the filters to an input image (i.e., at each layer, the feature map is the output of that layer). Think of it as (higher level) representations of the input. The feature map(s) is/are the output image(s) of each convolutional layer(s). The resultant number of feature maps equals the number of filters.

**Data augmentation (DA)** [124]:

ML (especially DL) model performance often improves with an increase in the amount of data. The common case in most ML/DL applications, especially in image classification tasks, is that obtaining new training data is not easy. Thus, we need to make good use of the existing (relatively small) training set. DA is one technique to expand the training dataset from existing training data in order to improve the performance and generalizability of DL models. DA enriches (i.e., “augments”) the training data by creating new examples through random transformation of existing ones. This way, we artificially boost the size of the training set, reducing overfitting. Thus, to some extent, DA can also be viewed as a regularization technique.

Image DA is perhaps the most well-known type of DA and involves creating transformed versions of images in the training dataset that belong to the same class as the original image. The ultimate goal is to expand the training dataset with new, plausible examples (i.e., variations of the training set images that are most likely to be seen by DL models). For example, a horizontal flip of a bike photo may make sense, because the photo could be taken from the left or right. A vertical flip of a bike image does not make sense and would probably not be appropriate as the model is very unlikely to see a picture of an upside down bike. Transformations for image DA include a range of operations from the field of image manipulation (e.g., rotation, shifting, resizing, flipping, zooming, exposure adjustment, contrast change, and much more). This way, a lot of new samples can be generated from a single training example.

Note that image DA is typically only applied to the training dataset, and NOT to the validation or test dataset. This is different from data preparation such as image resizing and pixel scaling; those must be performed consistently across all datasets that interact with the model. The choice of the specific DA techniques used for a training dataset must be chosen carefully and within the context of the training dataset and knowledge of the problem domain. It can be useful to experiment with DA methods in isolation and in concert to see if they result in a measurable improvement to model performance, perhaps with a small prototype dataset, model, and training run.

**DeepLabV3+** [125]: DeepLabV3 was firstly proposed to enable deep CNNs to segment features in images *at multiple scales.* ResNet-50 and ResNet-101, two variations on the popular residual network (ResNet) architecture, are the tested backbones for DeepLabV3. Through the use of residual blocks, atrous convolution, and a spatial pyramid pooling module, the authors showed that their new architecture achieved comparable performance to other SOTA models in image segmentation tasks without the need for further post-processing. The authors further improved DeepLabV3 and named the new version DeepLabV3+ [126], which combines atrous spatial pyramid pooling modules with an encoder–decoder module. This further improved the performance of DeepLabV3 while sharpening predicted feature boundaries. The DeepLabV3+ architecture is very popular in the water body extraction literature.

**Generative adversarial network (GAN):** GAN is a class of unsupervised DL frameworks in which two neural networks compete with each other. One network, the generator, tries to create synthetic or false images which fool the discriminator network. The discriminator, in turn, attempts to discern which images coming from the generator are actual vs. synthetic images [127]. GANs use a cooperative zero-sum game framework to learn. Among many variants of GAN, cycleGAN [128] is a technique for training unsupervised image translation models using the GAN architecture and unpaired collections of images from two different domains. CycleGAN has been demonstrated on a wide range of applications, including season translation, object transfiguration, style transfer, and generating photos from paintings.

**Generalization, overfitting, underfitting and regularization** (referenced [123,129,130]):

The prediction results of an ML/DL model sit somewhere between (a) low-bias, low-variance, (b) low-bias, high-variance, (c) high-bias, low-variance, and (d) high-bias, high-variance. A low-biased, high-variance model is called overfit and a high-biased, low-variance model is called underfit. A trained model achieves the best performance, through generalization, when the best trade-off between underfitting and overfitting is found. Learning with good accuracy is good, but generalization is what matters most. A good model is supposed to have both low bias and low variance. Overfitting and underfitting should both be avoided, where regularization may help.

Generalization: In ML/DL, generalization refers to the ability of a trained ML/DL model to react to new (i.e., previously unseen) data, drawn from the same distribution as the training data used to create the model. That is, after being trained on a training set, an ML/DL model can digest new data and make accurate predictions. The generalizability of an ML/DL model is central to the success of that model.

Overfitting vs. underfitting: Variance and bias are two important terms in ML. Variance refers to the variety of predicted values made by an ML model (target function). Bias means the distance of the predictions from the actual (true) target values. A high-biased model means its prediction values (average) are far from the actual values. In addition, high-variance prediction means the prediction values are highly varied.

If an ML/DL model has been trained too well on training data, it will be unable to generalize. It will make inaccurate predictions when given new data, making the model useless even though it is able to make accurate predictions for the training data. This is called overfitting. Underfitting happens when a model has not been trained enough on the data. Underfitting models are not useful either, as they are not capable of making accurate predictions, even with the training data.

Low error rates and a high variance are good indicators of overfitting. To avoid overfitting, part of the training dataset is typically set aside as the “test set” to check whether a trained model is overfitting. If the training data has a low error rate and the test data has a high error rate, it signals overfitting. An overfit model would have very low training error on seen training data but very high error from unseen datasets (e.g., testing dataset and new datasets beyond training and testing data). This is because the model maps the training set perfectly and any deviation from the training set would result in errors. An underfit model has high training error in training data and testing error in testing data and thus in new unseen data. This is because the model cannot generalize the training data correctly. Thus, the model will have a very high training error.

Regularization (also known as shrinkage): When an ML/DL model becomes too complex, it is most likely to suffer from *overfitting*. To avoid overfitting, regularization is a collection of methods to constrain and make an ML/DL model simpler and less flexible. Specifically, regularization methods are used to avoid high variance (i.e., bias/underfitting) and overfitting and thus to increase generalization. Intuitively, it follows that the function the model represents is simpler, less unsteady. Thus, predictions are smoother, and overfitting is less likely. Certain approaches are applied to different ML algorithms, for example, pruning for DT, dropout techniques for NN, and adding a penalty parameter to the cost function in regression.

**Google Earth (GE):** GE is a computer software, formerly known as Keyhole EarthViewer, that renders a 3D representation of Earth based primarily on satellite imagery. It has a web version at https://earth.google.com/web/, accessed on 2 January 2022. Since GE version 4.3, Google fully integrated Street View into Google Earth. Street View displays 360° panoramic street-level photos of select cities and their surroundings. The photos were taken by cameras mounted on automobiles, can be viewed at different scales and from many angles, and are navigable by arrow icons imposed on them.

**Google Earth Engine (GEE) and Microsoft Planetary Computer (MPC):**

GEE and MPC share similar goals (e.g., cloud storage and computing support for geospatial datasets), but have their own primary focus. For example, GEE is the pioneer in the area of RS cloud computing (launched in 2010, has 495 datasets in total as of 22 December 2021), and MPC, launched in 2020 (contains 17 datasets in total as of 22 December 2021), with a primary focus on climate change and sustainable environmental studies.

GEE [131,132]: GEE is a cloud-based platform for planetary-scale geospatial analysis, launched in 2010 by Google. GEE combines a multipetabyte catalog of satellite imagery and geospatial datasets with planetary-scale analysis capabilities. Scientists, researchers, and developers use GEE to detect changes, map trends, and quantify differences on the Earth’s surface. GEE brings Google’s massive computational capabilities to bear a variety of high-impact societal problems (e.g., deforestation, drought, disaster, disease, food security, water management, climate monitoring, and environmental protection). GEE has been available for commercial use from 2021 and remains free for academic and research use.

MPC [133,134]: The world lacks comprehensive, global environmental data. Microsoft Chief Environmental Officer (CEO), Dr. Lucas Joppa, imagines an international database that would provide the world with “information about every tree, every species, all of our natural resources”. Microsoft President Brad Smith further emphasized that “it should be as easy for anyone in the world to search the state of the planet as it is to search the internet for driving directions or dining options”, and Microsoft believes technology and AI is the key to get there, in hopes that this information will allow people to “come together and solve some of the greatest environmental and sustainability challenges we face today”.

To support sustainability decision-making with the power of cloud computing and AI, similar to GEE, since December 2020, Microsoft is using ML and computing power to aggregate global environmental data (contributed by individuals around the world coupled with machinery placed in water, space, land, and air environments) into a planetary computer for a sustainable future. MPC, described as a “global portfolio of applications connecting trillions of data points”, is designed to use AI to synthesize environmental data into practical information regarding the Earth’s current ecosystems. For the first time, there will be a concise and comprehensive compendium of international ecosystem data. Not only will this allow for essential environmental information to be readily available to individuals across the world, but the planetary computer will predict future environmental trends through ML. In short, MPC integrates a multipetabyte catalog of global environmental data with APIs, a flexible scientific computing environment that allows people to answer global questions about that data, and applications that place those answers in the hands of conservation stakeholders.

**Image classification:** The concept of image classification in RS and ML/DL settings has different meanings. In RS research, the image classification is at pixel level (this is what semantic segmentation does in CV, ML, and DL settings; see the concept definition below). In contrast, in an ML and DL setting, image classification does not refer to assigning each individual pixel to a class (e.g., vegetation, water), but rather to assign the entire image to a specific class (e.g., flooded vs. not flooded) [135].

**Instance segmentation:** Unlike semantic segmentation, instance segmentation identifies each object instance of each pixel for every known object within an image. Thus, labels are instance-aware. Instance segmentation is essential to tasks such as counting the number of objects and reasoning about occlusion.

**Normalized difference moisture index (NDMI)** [136,137]: Normalized difference moisture index (NDMI) is a satellite-derived index from the near-infrared (NIR) and short wave infrared (SWIR) channels of RS imagery (note that some literature used NDMI interchangeably with NDWI; check the NDWI entry in this Appendix B for clarification).

NDMI is sensitive to the moisture levels in vegetation, and thus used to determine vegetation water content. It can be used to monitor droughts as well as monitor fuel levels in fire-prone areas. NDMI uses NIR and SWIR bands to create a ratio designed to mitigate illumination and atmospheric effects. It is calculated as a ratio between the NIR and SWIR values from RS imagery, see the formula below. For example, in Landsat 4–7, NDMI = (Band 4 − Band 5)/(Band 4 + Band 5). In Landsat 8, NDMI = (Band 5 − Band 6)/(Band 5 + Band 6). Delivered NDMI is a single band image. Similar to NDVI, NDMI values are between −1 and 1.

NDMI = (NIR − SWIR)/(NIR + SWIR)

**Normalized difference vegetation index (NDVI)** [138]: NDVI is a pixel-wise mathematical calculation rendered on an image. It is an indicator of plant health, calculated by comparing the values of absorption and reflection of red and near-infrared (NIR) light. A single NDVI value can be determined for every pixel in an image, ranging from an individual leaf to a 500-acre wheat field, depending on the RS imagery resolution.

NDVI = (NIR − Red)/(NIR + Red)

NDVI values always fall between −1 and 1. Values between −1 and 0 indicate dead plants, or inorganic objects (e.g., water surfaces, manmade structures such as houses, stones/rocks, roads, clouds, snow). Bare soil usually falls within 0.1–0.2 range; and plants will always have positive values between 0.2 and 1 (1 being the healthiest plants). Healthy, dense vegetation canopy should be above 0.5, and sparse vegetation will most likely fall within 0.2 to 0.5. However, it is only a rule of thumb and we should always take into account the season, type of plant, and regional peculiarities to meaningfully interpret NDVI values.

**Normalized difference water index (NDWI) and modified NDWI (MNDWI) [139,140,141]**: The NDWI is an RS-based indicator sensitive to the change in the water content of leaves or water content in water bodies (detailed below). There are two versions of NDWI.

One was defined to monitor changes in water content of leaves, using near-infrared (NIR) and short-wave infrared (SWIR) wavelengths, proposed by Gao in 1996 [139] (to avoid confusion of the two versions of NDWI, this version is also called NDMI, see NDMI entry in this Appendix B).

NDWI = (NIR − SWIR)/(NIR + SWIR)

The other version of NDWI, proposed by McFeeters in 1996, was defined to monitor changes related to water content in water bodies, using green and NIR wavelengths [140]. The calculation formula is given below. It is obvious that the NDWI in the papers we reviewed in this article is the version of water content in water bodies. Modification of normalized difference water index (MNDWI) was proposed [141] for improved detection of open water by replacing NIR spectral band with SWIR.

NDWI = (Green − NIR)/(Green + NIR)

**PyTorch [142]**: PyTorch is an open-source deep learning framework developed and maintained by Facebook Artificial Intelligence Research (FAIR). At its core, PyTorch is a mathematical library that performs efficient computation and automatic differentiation on graph-based models. Achieving this directly is challenging, although thankfully, the modern PyTorch API provides classes and methods that allow you to easily develop a suite of deep learning models.

**Random forest (RF):** It is an ML (particularly, ensemble learning) algorithm that can be used for both continuous (regression) and categorical (classification) tasks [143]. RF is widely accepted as an efficient ensemble approach for land cover classification using RS data. It handles imbalanced data, missing values, and outliers well [144].

**Semantic segmentation:** In contrast to instance segmentation, semantic segmentation aims to predict categorical labels for each pixel for every known object within an image, without differentiating object instances [145]. Thus, its labels are class-aware.

**Support vector machine (SVM):** SVM is a (supervised) machine learning algorithm that provides solutions for both classification and regression problems. The support-vector clustering [146] algorithm applies the statistics of support vectors (developed in the support vector machine algorithm) to categorize unlabeled data and is one of the most widely used clustering algorithms in many applications.

**TensorFlow:** TensorFlow is an open-source deep learning framework developed and maintained by Google. Although using TensorFlow directly can be challenging, the modern tf.keras API brings the simplicity and ease of use of Keras to the TensorFlow project.

**Transfer learning (TL):** TL is one powerful technique that makes learning in (deep) ML transferable. TL was initially proposed in [147] and recently received considerable attention due to recent significant advances in DL [123,148,149,150,151,152]. Inspired by humans’ capabilities to transfer knowledge across domains (e.g., the knowledge gained while learning violin can be helpful to learn piano faster), TL aims to leverage learned knowledge from a related domain to achieve a desirable learning performance with minimized number of labeled samples in a target domain [151]. The main idea behind TL is that it is more efficient to take a DL model trained on an (unrelated) massive image dataset (e.g., ImageNet [87]) in one domain, and transfer its knowledge to a smaller dataset in another domain instead of training a DL classifier from scratch [153], as there are universal, low-level features shared between images for different problems.

**U-Net:** CNNs gave decent results in easier image segmentation problems but have not made any good progress on complex ones. This is where UNet comes in. UNet was first designed especially for medical image segmentation in [154]. It demonstrated such good results that it was used in many other fields afterwards. UNet is an improved architecture developed for biomedical image segmentation [154]. The UNet architecture stems from a fully convolutional network (FCN) first proposed by Long and Shelhamer in [155] and its architecture was modified and extended to work with fewer training images and to yield more precise segmentations. The architecture of UNet resembles a “U”, which justifies its name.

The UNet architecture includes three sections: the contraction, the bottleneck, and the expansion section. The bottommost layer mediates between the contraction layer and the expansion layer. The number of expansion blocks is the same as the number of contraction blocks. Most importantly, UNet uses a novel loss weighting scheme for each pixel such that there is a higher weight at the border of segmented objects. Specifically, all pixel-wise softmax applied on the resultant image is followed by a cross-entropy loss function. Each pixel is classified into one of the classes. The idea is that even in segmentation, every pixel has to lie in some predefined category. Thus, a segmentation problem was converted into a multiclass classification and it performed very well compared to the traditional loss functions.

## Appendix C. Common Evaluation Metrics in AI/ML/DL/CV Classification and Regression, and Segmentation Tasks

Many evaluation criteria have been proposed and are frequently used to assess the performance of AI/ML/DL/CV models. No single evaluation metric can tell a full story of a trained model. To better select appropriate evaluation metrics for certain domain problems and tasks, in this appendix, we provide brief definitions to some commonly used evaluation metrics (ordered alphabetically; referenced [123,129,130,156,157]) in AI/ML/DL/CV for classification, regression, and segmentation tasks in our review (i.e., those listed in the field of “Evaluation metrics” in Table 2 and Table 3). For readability, we group some related metrics together. In the following formulas, TP refers to true positive, FP to false positive, FN to false negative, and TN to true negative. TP samples are those that are in the positive category and are correctly predicted as positive. FPs are not annotated as the positive category but are incorrectly predicted as positive. TNs are correctly predicted as negative, while FNs are predicted as negative when they are actually labeled as positive.

**Accuracy, overall accuracy (OA), commission error (CE), omission error (OE), producer’s accuracy (PA), user’s accuracy (UA), and pixel accuracy (PixA)** [31,156,158,159,160,161]:

To better understand the metrics in this group, let us use the same confusion matrix shown below in Figure A2 to calculate the accuracy metrics in this group. Confusion matrix, also called error matrix, is a table that allows us to visualize the performance of a classification algorithm by comparing the predicted value of the target variable with its actual value [162].

(Average) Accuracy: Classification accuracy is the number of correct predictions made as a ratio of all predictions made. Accuracy with a binary classifier is measured as the following:

Accuracy (for binary classifier) = *(TP + TN)/(TP + TN + FP + FN)*

Note, however, that (average) accuracy for a multiclass classifier is calculated as the average of each accuracy per category (i.e., sum of accuracy for each category/number of categories) (see the definition and examples of binary classification and multiclass classification in Appendix A4 in [84]). For the example confusion matrix shown in Figure A2 (it is a multiclass classification problem), the (average) accuracy is calculated as follows:

(average) accuracy = (21/27 + 31/37 + 22/31)/3 = 77.5%

Accuracy is perhaps the most common evaluation metric for classification problems, and it is also the most misused. It is really only meaningful and appropriate when there are an equal number of observations in each category and that all predictions and prediction errors are equally important, which is often not the case. Accuracy alone cannot tell a full meaningful story of the ML/DL models, especially when a dataset encounters a severe data imbalance problem (detailed in [86]); other metrics, such as F-score, need to tell whether an ML/DL is not suffering from overfitting when the trained model has very high accuracy.

OA: It essentially tells us out of all of the samples what proportion were classified correctly. OA is usually expressed as a percent, with 100% accuracy being a perfect classification where all samples were classified correctly. OA is the easiest to calculate and understand but ultimately only provides very basic accuracy information. OA is formally defined as follows, where N is the number of total samples. OA calculation from the example confusion matrix in Figure A2 is (21 + 31+ 22)/95 = 74/95 = 77.9%

OA = *Number of correctly classified samples/N = (TP + TN)/N*

OE [31]: Errors of omission refer to samples that were left out (or omitted, as its name implies) from the correct category in the classified results. An example of OE is when pixels of a certain thing (such as maple trees), are not classified as maple trees.

OE is sometimes also referred to as a type II error (false negative). An OE in one category will be counted as a CE in another category. OEs are calculated by reviewing the reference sites for incorrect classifications. In the example confusion matrix shown in Figure A2, this is carried out by going down the columns for each category and adding together the incorrect classifications and dividing them by the total number of samples for each category. A separate OE is generally calculated for each category, as this will allow us to evaluate the classification accuracy and error for each category. OE is the inverse of the PA (i.e., OE = 1 − PA).

OE example based on the confusion matrix shown in Figure A2:

Water: Incorrectly classified reference sites: 5 + 7 = 12. Total # of reference sites = 33.

OE = 12/33 = 36%

Forest: Incorrectly classified reference sites: 6 + 2 = 8. Total # of reference sites = 39.

OE = 8/39 = 20%

Urban: Incorrectly classified reference sites: 0 + 1 = 1. Total # of reference sites = 23.

OE = 1/23 = 4%

CE [31]: Errors of commission are in relation to the classified results. An example of an CE is when a pixel predicts the presence of a feature (such as trees) and, in reality, it is absent (no trees are actually present). CE is sometimes also referred to as a type I error (false positive). CEs are calculated by reviewing the classified sites for incorrect classifications. This is performed by going across the rows for each class and adding together the incorrect classifications and dividing them by the total number of classified sites for each class. CE is the inverse of the UA (i.e., CE = 1 − UA). This makes sense and is easy to interpret, as when the predicted results are very reliable (with high UA score), the classification error would be low.

CE example based on the confusion matrix shown in Figure A2:

Water: Incorrectly classified sites: 6 + 0 = 6. Total # of classified sites = 27.

CE = 6/27 = 22%

Forest: Incorrectly classified sites: 5 + 1 = 6. Total # of classified sites = 37.

CE *=* 6/37 = 16%

Urban: Incorrectly classified sites: 7 + 2 = 9. Total # of classified sites = 31.

CE = 9/31 = 29%

PA: Similar to UA, PA is category-level-based accuracy. PA is the accuracy from the point of view of the “producer”. PA tells us how often real features in the ground truth are correctly shown in the classified results, or the probability that a certain ground truth category is classified as such. PA is formally defined as the following and is complement of the omission error (OE). PA = 100% − OE.

*PA = Number of correctly classified reference samples for a particular category/Number of samples from reference (i.e., annotated) data for that category = 1 − omission error*

PA example based on the example confusion matrix in Figure A2:

PA for water category = Correctly classified reference sites for water category/Total # of reference sites for water category = 21/33 = 64%.

PA for forest category = Correctly classified reference sites for forest category/Total # of reference sites for water category = 31/39 = 80%.

PA for urban category = Correctly classified reference sites for urban category/Total # of reference sites for uran category = 22/23 = 96%.

UA: Similar to PA, UA is category-level-based accuracy. UA is the accuracy from the point of view of a “user”, not the “producer”. UA essentially tells us how often the classified category will actually align with the ground truth. This is referred to as reliability (memory tip: users often care about reliability). The UA is a complement of the commission error (i.e., UA = 100% − Commission Error). UA is defined as the following:

UA = *Number of correctly classified samples for a particular category/Number of samples classified (i.e., predicted) to that category* = 1 − *commission error.*

UA example based on the example confusion matrix in Figure A2:

UA for *water* category = *Correctly classified sites for water category/Total # of classified sites for water category* = 21/27 = 78%.

UA for *forest* category = *Correctly classified sites for forest category/Total # of classified sites for water category* = 31/37 = 84%.

UA for *urban* category = *Correctly classified sites for urban category/Total # of classified sites for uran category* = 22/31 = 70%.

PixA [158]: Pixel accuracy is perhaps the easiest to understand metric conceptually. It is the percent of pixels in the image that are classified correctly. It is the simplest metric, simply computing a ratio between the amount of properly classified pixels and the total number of pixels. See the PixA calculation formula below, where N represents the total number of pixels in the assessment image, which equals *TP + TN + FP + FN*. *TP* denotes the number of target-pixels that were correctly detected, *FN* denotes the number of water body pixels not classified, *FP* is the number of nontarget pixels classified, and *TN* is the number of nontarget pixels classified as nontarget pixels. This metric can sometimes provide misleading results when the category representation is small within the image, as the measure will be biased in mainly reporting how well the classifier identifies negative category (i.e., where the category we care about, such as the water body category, is not present).

PixA = (*TP* + *TN*)/*N*

Edge overall accuracy (EOA), edge commission error (ECE), and edge omission error [33]:

The authors in [33] defined a few evaluation metrics for water edge pixel extraction accuracy. See the following steps for how these metrics are computed.

1. Manually draw the boundary of water body.
2. Apply morphological expansion to the water body boundary from step (1) to create a buffer zone, which is centered on the boundary line (radius = three pixels).
3. Finally, the pixels in the buffer area are judged.

Let the total number of pixels in the buffer area be M, the number of correctly classified pixels be M_R_, the number of missing pixels be M_O_, and the number of false alarm pixels be M_C_. EOA, EOE, and ECE are defined as below:

EOA = M_R_/M × 100%

EOE = M_o_/M × 100%

ECE = M_c_/M × 100%

**Intersection over union (IoU), mean intersection over union (mIoU), and frequency weighted intersection over union (FWIoU):**

In the formal definitions below, TP, TN, FP, and FN are the number of true positive, true negative, false positive, and false negative samples, respectively.

IoU [163,164]: It is the most popular and simple evaluation metric for object detection and image segmentation used to measure the overlap between any two shapes such as two bounding boxes or masks (e.g., ground-truth and predicted bounding boxes). Values of IoU lie between 0 and 1, where 0 means two boxes do not intersect and 1 indicates two boxes completely overlap. If the prediction is completely correct, IoU = 1. The lower the IoU, the worse the prediction.

mIoU [43]: It is a common evaluation metric for semantic image segmentation, which first computes the IOU for each semantic class and then computes the average over classes. The formula is given below.

mIoU = *TP*/(*TP* + *FP* + *FN*)

FWIoU [46,158]: It is an improvement over mIoU. As its name implies, it weights each class importance depending on their appearance frequency. The formal definition of FWIoU is given below, where n is the number of categories.

$$\mathrm{FWIoU}=\frac{1}{n+1}\sum_{i=0}^{n}\;\left(\frac{TP_{i}}{TP_{i}+TN_{i}+FN_{i}}\;\times \;\frac{TP_{i}+FN_{i}}{TP_{i}+FP_{i}+TN_{i}+FN_{i}}\right)$$

**Kappa statistic** [156,159,165,166,167,168,169,170,171,172]:

Kappa (aka Cohen’s kappa) statistic, a statistic that is frequently used to measure inter-annotator reliability (i.e., agreement) and also intra-annotator reliability for qualitative (i.e., categorical) items, is a very useful, but underutilized, metric. The importance of rater reliability is important because it represents the extent to which the data collected in a study are correct representations of measured variables. Note that this measure is to compare labeling by different human annotators, not a classifier versus a ground truth.

Cohen’s kappa statistic is a very good measure that can handle both multiclass and imbalanced class problems very well. In ML, for a multiclass classification problem (see Appendix A4.2 in [84] for the definition and other types of classification tasks), measures such as accuracy, precision, or recall do not provide the complete picture of the performance of a classifier. In addition, for imbalanced class problems (see section II.D Imbalanced data in [86] for details about data imbalance), measures such as accuracy are misleading, so measures such as precision and recall are used. There are different ways to combine the two, such as the F-measure, but the F-measure does not have a very good intuitive explanation, other than it being the harmonic mean of precision and recall.

The kappa statistic can be calculated by the following formula, where Pr(a) represents the actual observed agreement, and Pr(e) represents expected (i.e., estimated) chance agreement). Thus, Pr(a) = OA.

Kappa Statistic = (Pr(a) − Pr(e))/(1 − Pr(e))

Note that the sample size consists of the number of observations made across which raters are compared. Cohen specifically discussed two raters in his papers. The kappa is based on the chi-square table, and the Pr(e) is obtained through the following formula [166], where: cm^1^, cm^2^, rm^1^, rm^2^ represent column 1 marginal, column 2 marginal, row 1 marginal, row 2 marginal, respectively, and *n* represents the number of observations (not the number of raters).

$$\mathrm{Expected}\;(\mathrm{Chance})\;\mathrm{Agreement}=\frac{\left(\frac{{\mathrm{cm}}^{1}\times {\mathrm{rm}}^{1}}{n}\right)+\left(\frac{{\mathrm{cm}}^{2}\times {\mathrm{rm}}^{2}}{n}\right)}{n}$$

Similar to most correlation statistics, the kappa score can range from −1 to +1. Scores above 0.8 are generally considered good agreement; zero or lower mean no agreement (practically random labels). According to the scheme of [165], a value of <0 indicates no agreement, 0–0.20 is slight, 0.21–0.40 is fair, 0.41–0.60 is moderate, 0.61–0.80 is substantial, and 0.81–1 is almost perfect agreement.

Kappa is one of the most commonly used statistics to test interrater reliability, but it has limitations. Judgments about what level of kappa should be acceptable for health research are questioned. Cohen’s suggested interpretation may be too lenient for health-related studies because it implies that a score as low as 0.41 might be acceptable [166]. Additional measures have been proposed to make use of the kappa framework.

For example, in [159], the authors advocate against the use of kappa and proposed the alternative measures of quantity and allocation disagreement. Quantity disagreement (QD) is the disagreement between the classification and reference data resulting from a difference in proportion of categories. Allocation disagreement (AD) assesses a difference in the spatial location of categories. The two measures (i.e., QD and AD) sum to overall error (i.e., 1–OA).

**Mean Absolute Error (MAE) and Mean Absolute Percentage Error (MAPE)** [123,129,130,173]:

MAE: also called mean absolute deviation, MAE finds the average of the absolute differences between actual and predicted values. It gives an idea of how wrong the predictions were. MAE measure gives an idea of the magnitude of the error, but no idea of the direction (e.g., over- or underpredicting). MAE is defined as below [174], where *y_i_* is the actual true value, and *ŷ_i_* is the predicted value. MAE value lies between 0 to ∞. Small value indicates a better model, and a value of 0 indicates no error, or perfect predictions.

$$\mathrm{MAE}=\frac{1}{N}\sum_{i=1}^{N}\left|y_{i}-\hat{y_{i}}\right|$$

MAE is more robust to the outliers than MSE, as it is not sensitive to outliers. MAE treats larger and small errors equally. The main reason is that in MSE, through squaring the errors, the outliers, which usually have higher errors than other samples, obtain more attention and dominance in the final error and thus impact the model parameters. In addition, there is an intuitive maximum likelihood (MLE) interpretation behind MSE and MAE metrics. If we assume a linear dependence between features and targets, then MSE and MAE correspond to the MLE on the model parameters by assuming Gaussian and Laplace priors on the model errors, respectively.

MAPE [175]: MAPE, also known as mean absolute percentage deviation (MAPD), is the mean or average of the absolute percentage errors of forecasts. Error is defined as actual value (i.e., observed value) minus the forecasted value. Percentage errors are summed without regard to sign to compute MAPE. It is the most common measure used to forecast error and works best if there are no extremes to the data (and no zeros). Because absolute percentage errors are used, it avoids the problem of canceling positive and negative errors. The formula is given below, where *M* is mean absolute percentage error, *n* is number of times the summation iteration happens, *At* is the actual value, and *Ft* is the forecast value. The smaller the MAPE, the better the forecast.

$$M=\frac{1}{n}\sum_{t=1}^{n}\left|\frac{A_{t}-F_{t}}{A_{t}}\right|$$

**Precision, recall, sensitivity, specificity, and F-score** [156]:

Each measure in this group is a set-based measure [176]. The values of those measures are all from 0 to 1, with the best value at 1 and the worst score at 0.

Precision: The precision is mathematically defined by the following formula. Precision attempts to answer the question What proportion of positive identifications was actually correct? Precision refers to the proportion of the samples that is correctly classified amongst the samples predicted to be positive and is equivalent to user’s accuracy (UA) for the positive category, which is also equivalent to 1 − commission error.

Precision = *TP*/(*TP* + *FP*)

Recall (also called sensitivity or true positive rate): it refers to the proportion of the reference data for the positive category that is correctly classified and is equivalent to producer’s accuracy (also equivalent to 1 − omission error) for the positive category. It is calculated by the following formula. Recall attempts to answer the following question: What proportion of actual positives was identified correctly?

Recall = *TP*/(*TP* + *FN*)

Specificity (also called true negative rate): it refers to the proportion of negative samples that is correctly predicted and is equivalent to the producer’s accuracy (PA) for the negative category [177].

Specificity = *TN/(TN + FP)*

F-score (also called F1-score, F measure): Depending on the application domain, we may need to give a higher priority to recall or precision, but there are many applications where both recall and precision are important. Thus, it is natural to think of a way to combine these two metrics into a single one. One popular metric that combines precision and recall is called F1-score. The F1-score can be interpreted as a weighted harmonic mean of the precision and recall and is formally defined as below. There is always a trade-off between precision and recall of a model; if making the precision too high, we would see a drop in the recall rate, and vice versa.

F1-score = (2 × *Precision × Recall)/(Precision + Recall)*

The generalized version of F-score is defined as follows. F1-score is a special case of F_β when β = 1.

$$F_{\beta }=\left(1+\beta^{2}\right)\times \frac{precision\times recall}{\beta^{2}\times precision+recall}$$

**R^2^, mean squared error (MSE), root mean squared error (RMSE), and root mean squared logarithmic error (RMSLE)** [123,129,130,173]:

R^2^ is based on correlation between actual and predicted value; MAE is based on absolute value of error; MSE and RMSE are both based on square of error.

**R^2^:** R-squared, also known as the coefficient of determination, is a value between 0 and 1 that measures how well a regression line fits the data (i.e., indication of the goodness of fit of a set of predictions to the actual values in a regression model). The value range of R^2^ lies between 0 and 1 for no-fit and perfect fit, respectively. R^2^ is not sensitive to outliers.

The R-squared formula compares our fitted regression line to a baseline model. This baseline model is considered the “worst” model. The baseline model is a flat line that predicts that every value of y will be the mean value of y. R-squared checks to see if our fitted regression line will predict y better than the mean.

$$R^{2}=1-\frac{SS_{RES}}{SS_{TOT}}=1-\frac{\sum_{i}{\left(y_{i}-{\hat{y}}_{i}\right)}^{2}}{\sum_{i}{\left(y_{i}-\bar{y}\right)}^{2}}$$

*SS_RES_* refers to the residual sum of squared errors of the regression model; *y_i_* is the actual value, and *ŷ_ᵢ_* is the predicted value through the regression model. For example, if the actual y value was 58 but we had predicted it would be 47 then the residual squared error would be 121 and we would add that to the rest of the residual squared errors for the model.

*SS_TOT_* is the total sum of squared errors. This compares the actual y values to the baseline model (i.e., the mean). We square the difference between all the actual y values and the mean $\bar{y}$ and add them together.

MSE: MSE is perhaps the most popular metric used for regression problems. It essentially finds the mean (i.e., average) of the square of the difference (i.e., squared error) between actual and estimated values. Similar to MAE, MSE provides a gross idea of the magnitude of error. Let us assume we have a regression model that predicts the price of houses in the Boston area and let us say for each house we also have the actual price the house was sold for. The MSE can be calculated as the following, where *N* is the number of samples, *y_i_* is the actual house price, and *ŷ_i_* is the predicted value through the regression model. MSE value lies between 0 to ∞. Small value indicates a better model. Sensitive to outliers, it punishes larger errors more. MSE incorporates both the variance and the bias of the predicting model.

$$\mathrm{MSE}=\frac{1}{N}\;\sum_{i=1}^{N}{\left(y_{i}-\hat{y_{i}}\right)}^{2}$$

MSE measures how far the data are from the model’s predicted values, whereas R^2^ measures how far the data are from the model’s predicted values compared to how far the data are from the mean. The difference between how far the data are from the model’s predicted values and how far the data are from the mean is the improvement in prediction from the regression model.

RMSE: very straightforward, RMSE is the square root of MSE. Sometimes people use RMSE to have a metric with scale as the target values. Taking the house pricing prediction example, RMSE essentially shows what is the average deviation in your model predicted house prices from the target values (the prices the houses are sold for). Similar to MSE, RMSE value lies between 0 to ∞, with a small value indicating a better model. Similar to MSE, RMSE is sensitive to outliers and punishes larger errors more. The value of RMSE is always greater than or equal to MAE (RMSE >= MAE). The greater difference between them indicates greater variance in individual errors in the sample.

RMSLE: both RMSE and RMSLE are the techniques to find out the difference between the actual values and the predicted values by an ML/DL model. RMSLE is the root mean squared error of the log-transformed predicted and log-transformed actual values. RMSLE is formally defined as follows, where X denotes the predicted value and Y denotes the actual value, and n is the number of samples. Note that RMSLE adds 1 to both actual and predicted values before taking the natural logarithm to avoid taking the natural log of possible 0 (zero) values.

$$\mathrm{RMLSE}=\sqrt{\frac{1}{n}\;\sum_{i=1}^{n}{\left(log\left(x_{i}+1\right)-\log\left(\;y_{i}+1\right)\right)}^{2}}$$

RMSLE is very robust to outliers. When we compare the formula of the RMSE and RMSLE, the only difference is the log function. Basically, what changes is the variance measured. This small difference makes RMSLE much more robust to outliers than RMSE. In RMSE, outliers can explode the error term to a very high value, but in RMLSE, the outliers are drastically scaled down, therefore nullifying their effect.

RMSLE is often used when we do not want to penalize huge differences in the predicted and the actual values when both predicted and true values are huge numbers. (1) If both predicted and actual values are small: RMSE and RMSLE is same. (2) If either predicted or the actual value is big: RMSE > RMSLE. (3) If both predicted and actual values are big: RMSE > RMSLE (RMSLE becomes almost negligible).
